# Newly initiated carbon stock, organic soil accumulation patterns and main driving factors in the High Arctic Svalbard, Norway

**DOI:** 10.1038/s41598-022-08652-9

**Published:** 2022-03-18

**Authors:** T. Juselius, V. Ravolainen, H. Zhang, S. Piilo, M. Müller, A. Gallego-Sala, M. Väliranta

**Affiliations:** 1 Environmental Change Research Unit (ECRU), Ecosystems, Environment Research Programme, Faculty of Biological and Environmental Sciences, and Helsinki Institute of Sustainability Science (HELSUS), University of Helsinki, Viikinkaari 1, P.O. Box 65, 00014 Helsinki, Finland; 2 Fram Centre, Norwegian Polar Institute (NPI), 9296 Tromsø, Norway; 3 Key Laboratory of Cenozoic Geology and Environment, Institute of Geology and Geophysics, Chinese Academy of Sciences, Beijing, China; 4 Department of Geosciences and Geography, Faculty of Science, University of Helsinki, Yliopistonkatu 3, P.O. Box 4, 00014 Helsinki, Finland; 5 Geography, College of Life and Environmental Sciences, University of Exeter, Exeter, UK

**Keywords:** Palaeoecology, Wetlands ecology, Climate-change impacts

## Abstract

High latitude organic soils form a significant carbon storage and deposition of these soils is largely driven by climate. Svalbard, Norway, has experienced millennial-scale climate variations and in general organic soil processes have benefitted from warm and humid climate phases while cool late Holocene has been unfavourable. In addition to direct effect of cool climate, the advancing glaciers have restricted the vegetation growth, thus soil accumulation. Since the early 1900’s climate has been warming at unprecedented rate, assumingly promoting organic soil establishment. Here we present results of multiple organic soil profiles collected from Svalbard. The profiles have robust chronologies accompanied by soil property analyses, carbon stock estimations and testate amoeba data as a proxy for soil moisture. Our results reveal relatively recent initiation of organic soils across the Isfjorden area. The initiation processes could be linked to glacier retreat, and improvement of growing conditions and soil stabilization. Carbon stock analyses suggested that our sites are hot spots for organic matter accumulation. Testate amoebae data suggested drying of soil surfaces, but the reason remained unresolved. If continued, such a process may lead to carbon release. Our data suggest that detailed palaeoecological data from the Arctic is needed to depict the on-going processes and to estimate future trajectories.

## Introduction

The High Arctic areas are characterized by cold temperatures, short growing season, and low productivity. Areas not covered by ice are dominated by tundra with short vegetation. Due to the harsh climate, growth rates, abundancies and taxonomic richness are restricted and only the most suitable microclimatological locations support lush vegetation^1–3^. Yet, the organic soils of the high latitudes are a significant storage of organic carbon^4,5^. The formation of organic soils is controlled by the balance between the biomass production and decomposition. Under conditions with high productivity or low decomposition, biomass production may exceed the decomposition rate enabling accumulation of organic soil; typically, roughly 50% of mature peat is carbon^6^. It has been suggested that in the north, climate warming may markedly increase carbon sequestration of organic soils, as the improved growth conditions promote plant productivity^7^, moreover, palaeoecological evidence confirms the link between climate and organic soil development^6,8^. Since the last glacial epoch which ended ca. 12 ka. BP^9^ the climate in Svalbard has varied widely from relatively mild and humid during the early and mid-Holocene periods to cold and dry conditions during the late Holocene^10^. However, during these long-term climate periods, shorter shifts from the prevailing main climate trend have occurred^11,12^. The late Holocene cool period (4.2 ka BP to present), the so called Neoglacial era, was characterised by increased glacial activity and growth or emergence of glaciers, which were mostly absent during the earlier part of the Holocene^9,10,13–15^. As a direct consequence of glacial advance, prevailing peatlands and vegetated habitats were either buried under glaciers or re-located by moving ice masses, resulting in cessation of peat accumulation^12,15,16^. Nevertheless, these short- and long-term climate variations have provided alternating conditions for vegetation establishment and development simultaneously promoting accumulation of organic soils and peat.

The High Arctic Svalbard archipelago is well suited for studying recent organic soil and peat accumulation. Since the end of the ‘Little Ice Age’ (LIA), which persisted from ca. 1500 CE to the beginning of twentieth century^17^, temperatures in Svalbard have increased^18^. During the last 120 years, instrumental records show a 0.32 °C increase in annual mean temperatures per decade, which is ca. 3.5 times the increase of the global mean temperature for the same period^19^. This warming has been especially high, 1.7 °C per decade, during the last 30 years. In addition, precipitation has increased at a rate of 2% per decade since the early twentieth century, based on measurements at Svalbard Airport^20^. These ongoing climatic changes should also be mirrored in the organic soils and peat in Svalbard.

In addition to direct climate effects, organic soil development can be driven by other external factors, and many of these are very relevant for Svalbard. Presence of bird colonies have been recorded regularly near peat deposits in Svalbard^21–23^. The fertilizing effect of bird manure provides significant boost for local nutrient regime in the harsh High Arctic conditions, thus promoting vegetation growth and creating areas with higher plant biomass production^24–27^. If combined with suitable hydrology, these locations can be favourable for long-term peat and organic soil accumulation^28^. Moreover, herbivores, and reindeer particularly, can influence vegetation via grazing, trampling and manuring which can lead to the vegetation becoming dominated by a thick layer of mosses with interspersed graminoids and forbs^29–31^. While the sea bird colonies are concentrated on coastal cliffs, reindeer have occupied the whole land area of Svalbard^32^, also shallow mountain slopes where topographical conditions favour peat formation^33^.

Svalbard soils are heavily disturbed by glacial meltwaters and long-term presence of vegetation is required to stabilize the substratum and initiate effective organic soil accumulation^34^. After stabilization and initiation, soil development factors, such as mineral substrate type, topography and microtopography affect the development of vegetation and thus the organic matter accumulation^35,36^.

Northern carbon stocks are important landscape elements in global carbon budget^5,7^. External driving factors affecting carbon dynamics are manifold, and consequences unevenly dispersed across the landscape. Thus, multiple sites should be targeted to investigate organic soil accumulation patterns to achieve a comprehensive understanding of past and current trends and drivers. Due to the multitude of overlapping promoting and impeding factors, the future of the organic soils in the Arctic has remained unpredictable. Climate has been prominently warming over the last decades and this has been recorded also in the Svalbard, thus information on organic soil succession patterns from Svalbard may provide us important knowledge and insights for the High Arctic terrestrial processes in the future. Here we present a multiproxy study where we reconstructed organic soil accumulation initiation and development over the last centuries from multiple sites to capture the uneven development patterns and environmental drivers.

## Material and methods

### Study sites

**Figure 1:**
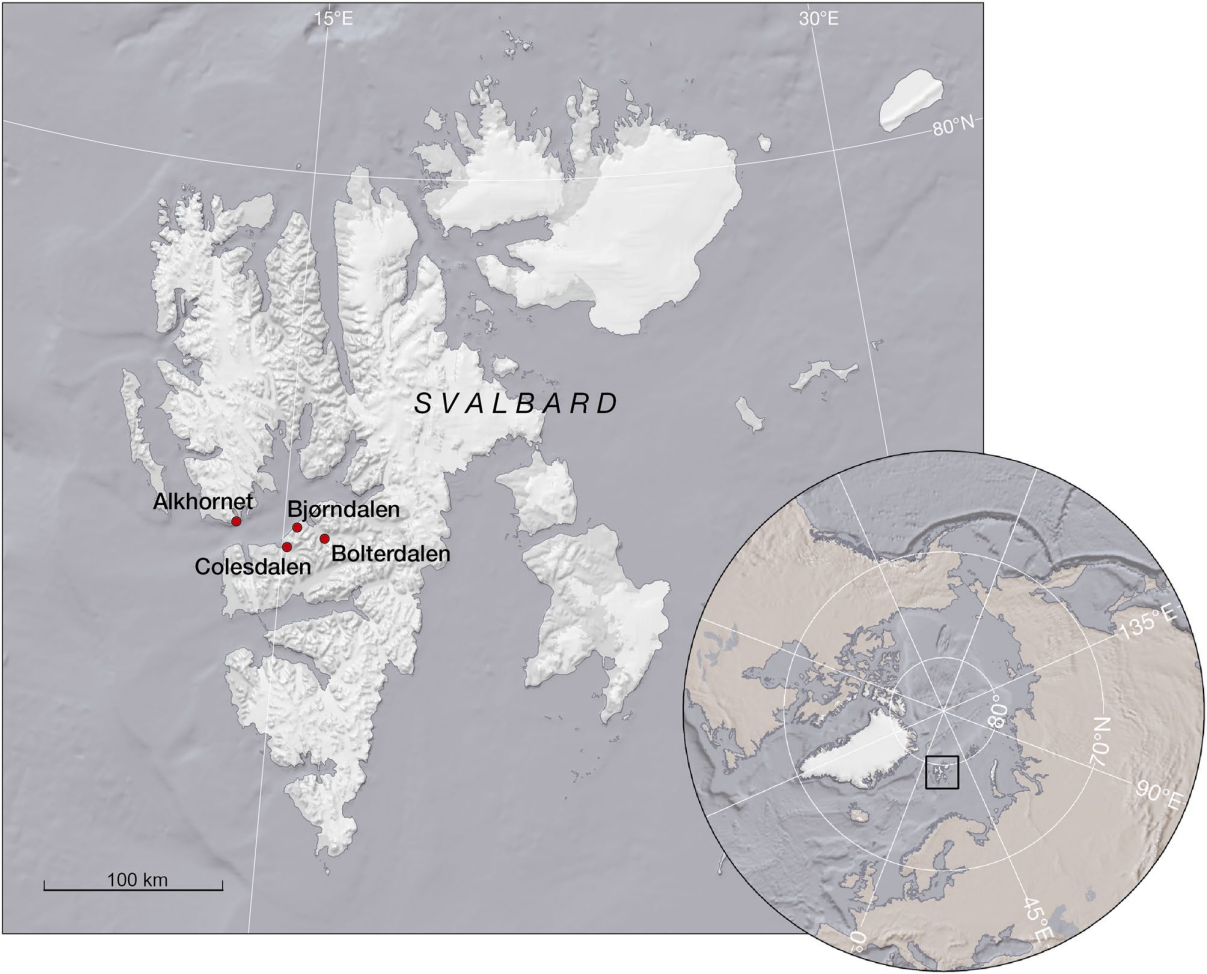
The Svalbard archipelago and location of our study sites in Spitsbergen, the main island of the Svalbard.

Svalbard is an archipelago in Norway, situated in the High Arctic between 74° and 81° northern latitudes (Fig. 1). The climate in Svalbard is determined not only by its high latitude but also by sea currents, sea-surface temperature, sea-ice extent, and prevailing wind conditions^37,38^. Land area in Spitsbergen, the largest island in the Svalbard is mostly covered by glaciers (~ 62%) and polar deserts (~ 22%), while vegetated terrain comprise of only of 8% of the land area^39^. Long-term presence of glaciers can be seen in the island’s topography, as it is deeply carved by fjords. Our study area surrounds Isfjorden, a large fjord located on the western part of the largest island of the archipelago, Spitsbergen.

**Figure 2:**
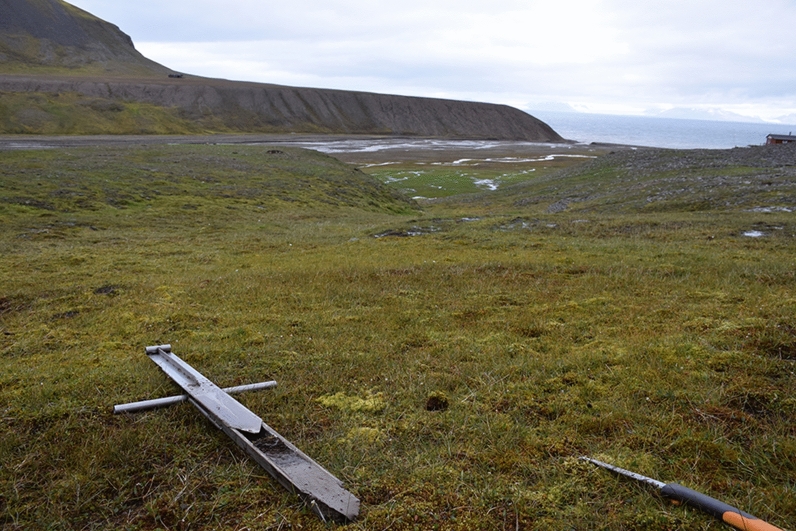
The general view of the Bjørndalen study site. The variations at the local scale can be seen in wet depression with mosses and dry surfaces with grass species.

**Figure 3:**
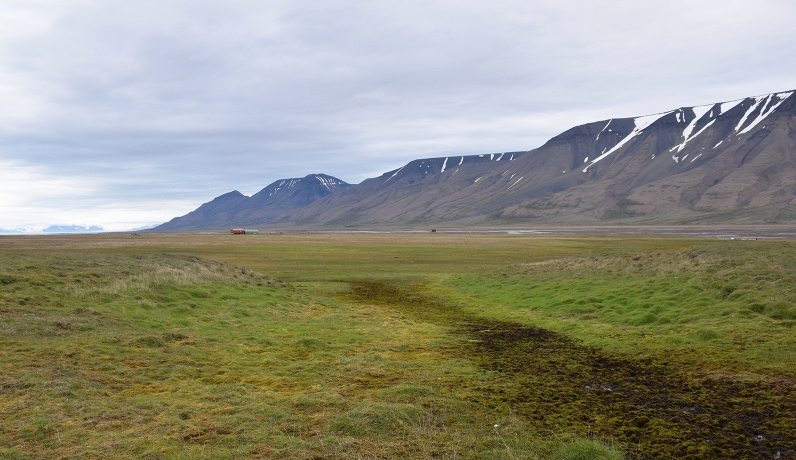
The general view of the Bolterdalen study site. Similar to Bjørndalen, variations between wet and dryer microforms of landscape can be seen.

**Figure 4:**
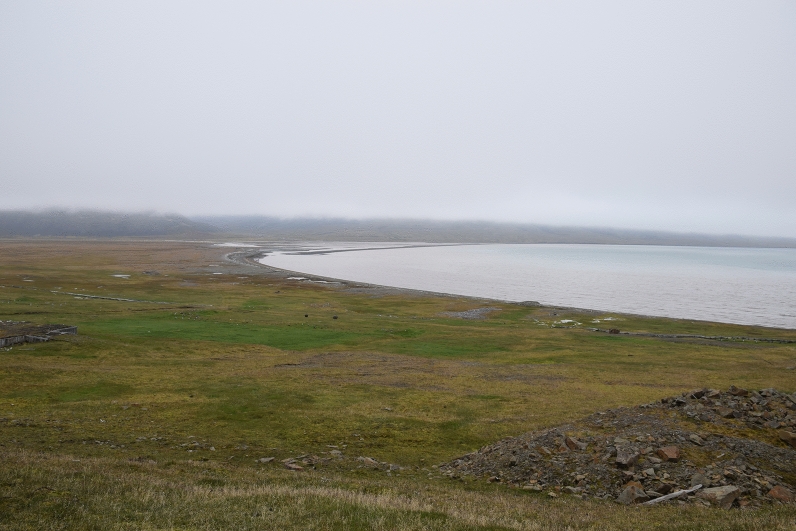
The general view of the Colesdalen study site. Vegetation and microforms in Colesdalen are similar to those of the Bjørndalen and Bolterdalen. In the front and at the back of the image non-vegetated mineral disturbance surfaces can be seen.

**Figure 5:**
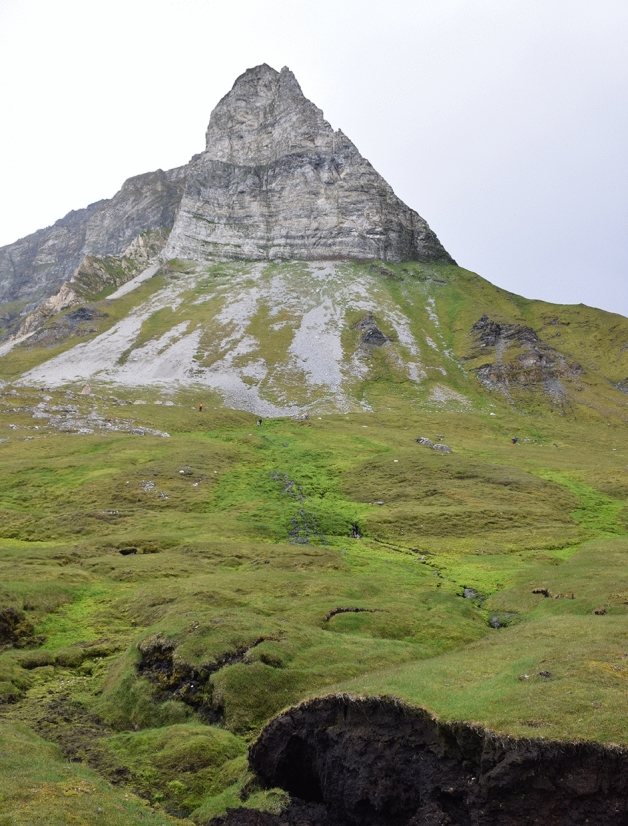
The general view of the Alkhornet study site. Thick peat layer can be seen in the wall of erosion gully on the front and bird cliff providing nutrients for the site in the back of the image.

**Figure 6:**
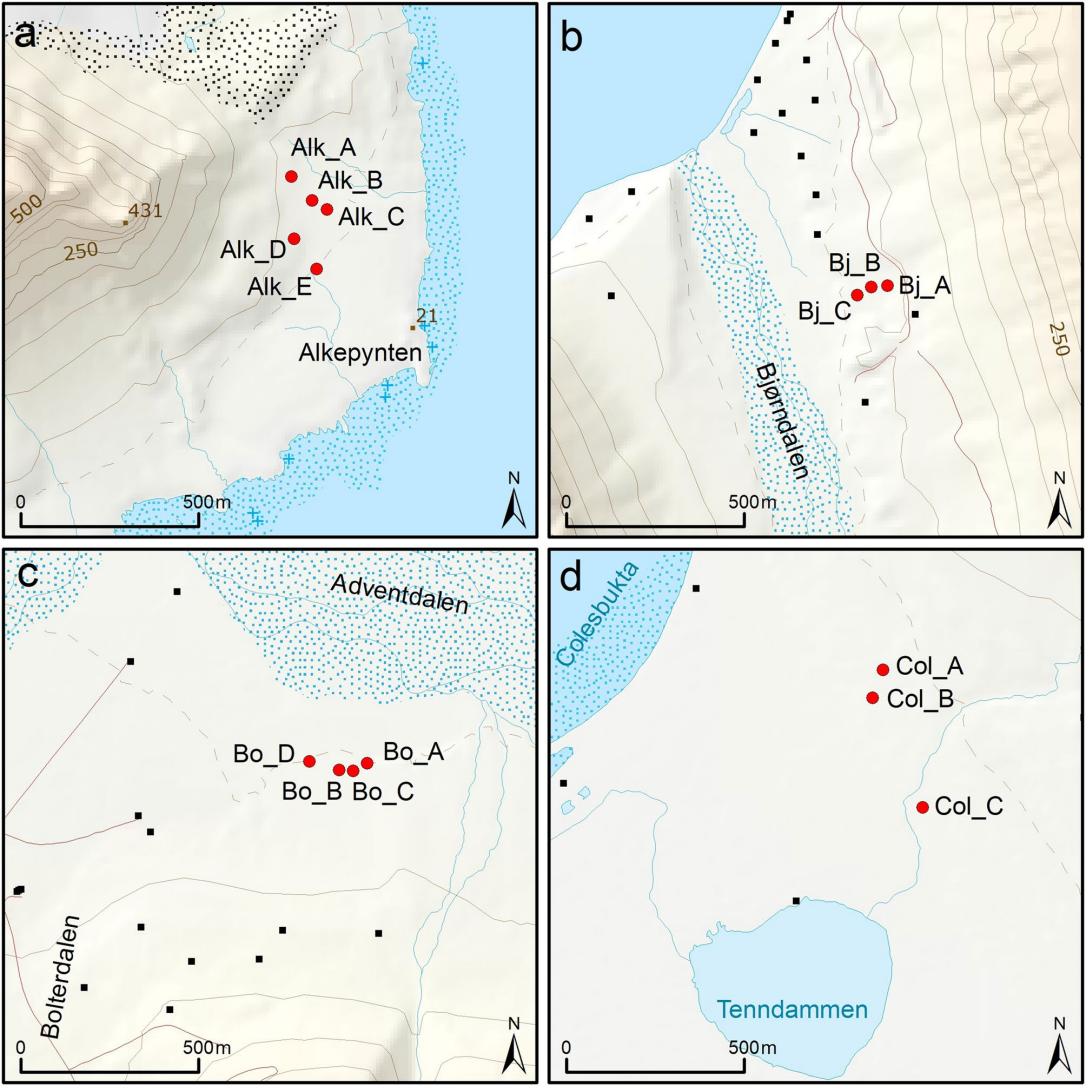
Study points of each study site. (2**a**) Alkhornet, (2**b**) Bjørndalen, (2**c**) Bolterdalen and (2**d**) Colesdalen.

Four study sites were included in this study (Table 1, Fig. 1). Bjørndalen (Bj) (Fig. 2), Bolterdalen (Bo) (Fig. 3) and Colesdalen (Col) (Fig. 4) represent sites with thin organic soils (Supplementary Figs. S1 and S2). In contrast, in Alkhornet (Alk) (Fig. 5), the peat layer was thicker (Supplementary Fig. S3 and S4). In total, 15 cores from these four sites were investigated (Table 1, Fig. 6a–d). The study site area (ha) was estimated using satellite imagery and vegetation map provided by Norsk Polarinstitut^40^.

**Table 1:** Study site information.

Study site	Study points	Study area, ha	Latitude, N	Longitude, E	Elevation (m, a.s.l.)	MAT (°C)	MAP (mm/year)
Bjørndalen	3	3.2	78°13.413ʹ	015°19.838ʹ	62–69	− 5.9	186
Bolterdalen	4	9.9	78°10.339ʹ	016°02.063ʹ	58–60		
Colesdalen	3	45.9	78°06.467ʹ	015°02.634ʹ	41–47		
Alkhornet	5	123.5	78°12.905ʹ	013°49.574ʹ	58–77

The vegetation at Bjørndalen, Bolterdalen and Colesdalen were classified as herbaceous moss tundra. The vegetation on these sites is dominated by various mosses, sedges and grasses (Supplementary data 1). In addition, *Salix polaris* dwarf shrubs are also found at these sites. *Sphagnum* mosses occur sporadically in Bjørndalen. In Colesdalen, at least 3 different *sphagnum* taxa were encountered, and they are more abundant than in Bjørndalen (Supplementary Fig. S5). No *sphagna* is found in Bolterdalen.

At Alkhornet, vegetation is similar to the other sites, but more grasses (*Poa*) are present. No *sphagna* were found within proximity of our study points at Alkhornet. The Alkhornet site is heavily influenced by the adjacent bird cliff and its fertilizing effect.

We acquired measured meteorological data for the last ca. 100 years from the Svalbard airport meteorological station^41^. Annual mean temperature (ºC) for period 1899–2019 and annual precipitation (mm/year) for period 1912–2019 from the Svalbard Airport composite series^19,41^ were used as the best available dataset for our study sites. For years 1942, 1943 and 1944 no precipitation data were available.

### Soil profile sampling

We investigated initiation and organic soil accumulation processes in four study sites, all including several study points. All together 15 soil profiles were collected from the four study sites in August 2018 with a box corer (7 × 4 × 65 cm) (Table 1, Fig. 6a–d). Three profiles each were collected from Bjørndalen, and Colesdalen and four from Bolterdalen. For these sites, sampling was performed down to the glaciogenic till subsoil underlaying the organic soil. In Alkhornet, five soil profiles were collected either down to the mineral subsoil (Alk_A) or to the depth of the permafrost (Alk_B, Alk_C, Alk_D, Alk_E). For each study site, vegetation was described (see Supplementary data 1) and water table depth (cm) and pH were measured. The sampling points represent the prevailing vegetation. The collected profiles were wrapped in plastic film and gutters to avoid compaction and frozen in the Longyearbyen city to − 80 °C for two nights to exterminate possible *Echinococcus* parasites. Samples were transported in PVC tubes to the University of Helsinki, where thawed profiles were sliced to 1 cm subsamples and stored in plastic bags at 6 °C. Same subsamples were used for all further analyses.

### Organic soil chronologies

For all profiles, we applied AMS radiocarbon (^14^C) determinations to date the basal subsamples of the studied soil profiles. A total of 16 subsamples (Table 2) from 15 cores were dated by AMS radiocarbon (^14^C) determination at the Finnish Museum of Natural History (LUOMUS, Helsinki, Finland) (7 samples) or at the Poznan Radiocarbon Laboratory (Poznan, Poland) (9 samples). To determine the organic soil initiation history, we dated the deepest basal layer that contained organic material overlying the mineral soil, chosen by visual determination using soil texture and colour (Supplementary Figs. S1–S5). In Alkhornet, no mineral contact was detected for 4 out of 5 study points and the bottom of the active layer was dated instead. One mid-profile sample was ^14^C dated for Alkhornet study point Alk_C to gain further understanding of development of deeper peat layers in Svalbard. Nine soil profiles (Alk_B Alk_C, Alk_E, Bj_A, Bj_C, Bo_B, Bo_C, Col_A, Col_B) were chosen for more extensive study, henceforth called the focus sections. For these profiles, basal ages were supplemented by ^210^Pb chronologies, carried out at the University of Exeter, UK, to capture the most recent deposition history. A small amount (of 0.12–0.56 g) of freeze-dried, ground material at 2 cm intervals was spiked with a ^209^Po yield tracer and analysed following the procedure described in Kelly et al.^42^ and Estop-Aragonés et al.^43^ ^14^C and ^210^Pb ages were combined to create age-depth models using PLUM version 0.1.5.1 package^44^ in R version 4.0.3^45^.

**Table 2:** Study site properties.

Study site	Study point	Laboratory	Lab. code	Core length, cm	pH	WTD, cm	Basal age, BP	± (1σ)	pMC(%)	± (1σ)	Basal age, cal. BP
Alkhornet	Alk_A	LUOMUS	Hela-4355	11	6	–	2005	23			1950
	Alk_B*	Poznań	Poz-108087	30	4,5	–	1510	35			1495
	Alk_C(1)	LUOMUS	Hela-4354	15	5	–	1195	22			1340
	Alk_C(2)*	Poznań	Poz-108088	25	5	–	5030	40			5865
	Alk_D*	LUOMUS	Hela-4356	24	5,5	10	3080	22			3220
	Alk_E*	Poznań	Poz-108089	32	4,5	–	4480	35			5230
Bjørndalen	Bj_A	Poznań	Poz-108080	13	5	6	MODERN		100,11	0,31	65
	Bj_B	LUOMUS	Hela-4358	10	5	8	> MODERN		129,31	0,34	− 25
	Bj_C	Poznań	Poz-108081	7	5	6	MODERN		106,98	0,33	− 35
Bolterdalen	Bo_A	LUOMUS	Hela-4359	12	5	–	MODERN		101,76	0,29	− 5
	Bo_B	Poznań	Poz-108082	13	5	–	MODERN		107,38	0,33	− 10
	Bo_C	Poznań	Poz-108083	16	5	8	MODERN		100,41	0,32	− 5
	Bo_D	LUOMUS	Hela-4360	9	5	–	MODERN		103,34	0,3	− 10
Colesdalen	Col_A	Poznań	Poz-108084	11	5	–	MODERN		128	0,37	− 30
	Col_B	Poznań	Poz-108085	11	5	32	MODERN		102,24	0,32	− 10
	Col_C	LUOMUS	Hela-4357	12	5	–	176	26			155

For study points marked with *, core length and basal age represent depth and age down to bottom of active layer. The study point Alk_C(1) is a mid-profile sample. For water table depth (WTD), water table was not reached on study points marked with -. The calibrated basal ages were acquired from Plum age-depth models.

### Organic soil properties analysis

Soil property analyses included loss-on-ignition (%), bulk density, nitrogen content and carbon content analyses. To determine the organic content of the profiles, loss on ignition (LOI) was measured at 1 cm resolution^46^. In addition, dry bulk density (BD, g cm^−3^) of every 1 cm subsample was calculated from weight and volume measurements. For the focus sections samples, carbon (C) and nitrogen (N) content was determined at 4 cm intervals using a LECO TruSpec micro Elemental Determinator at the University of Helsinki. From these values, a C/N ratio was calculated.

We calculated the amount of carbon (g C cm^−2^) for each individual study point to determine the total amount of stored carbon since the initiation determined by the basal age. This was calculated by multiplying BD (g cm^−3^) with carbon content (%) for each 1 cm layer of each study point. The amount of carbon for each layer was added together to have the total carbon storage down to the depth of the basal layer or to the bottom of the active layer (Alk_B, Alk_C, Alk_D and Alk_E). For focus sections, the measured carbon content (%) was used. For other sites, we used an average carbon content, calculated from the carbon content values of focus sections of corresponding study site.

### Testate Amoeba analysis

Testate amoeba analysis was conducted for the focus sections, excluding Alk_E, at 2 cm resolution. The cores were analysed to a depth ranging from 11–17 cm depending on the length of the core and the amount of specimen present. At depths greater than 17 cm no testate amoeba analysis was performed due to decay of testate amoebas affecting reliability of the method. Preparation of testate amoeba samples followed a modified version of the standard method^47^. Volumetric samples (ca. 2 cm^3^) were simmered in distilled water for 15 min and stirred occasionally. The samples were then sieved with a 300-μm mesh and back sieved with a 15-μm mesh. Materials retained on the 15-μm sieve were centrifuged at 3000 rpm for 5 min. 100 individual shells for each sample were counted and identified to species or “type” level under a light microscope at the magnification of 200–400. Taxonomy followed Charman et al.^48^ and was supplemented with online sources^49^. At least 50 specimens were counted in samples with low testate amoeba concentrations^50^; whenever this amount could not be reached, the samples were removed from further analyses.

Testate amoebae were categorized into six groups of hydrological preference, i.e. dry, dry-intermediate, intermediate, wet-intermediate, wet and wide/unclear^51–55^. No local or regional transfer function exists, so this method was not applied to the testate amoeba data.

## Results

### Chronology

Core-specific basal ages vary from 1795 cal yr CE (Col_C) to 1985 cal yr CE (Bj_B) in Bjørndalen, Bolterdalen and Colesdalen (Table 2). In Alkhornet, basal active layer ages are older ranging from 5865 cal yr BP (Alk_C) to cal yr 1495 BP (Alk_B) (Table 2). Alk_A, located at the margin of the study site, is the only study point in Alkhornet where the mineral subsoil was reached during sampling. The basal age for this study point is cal yr 1950 BP.

### Organic soil properties

**Figure 7:**
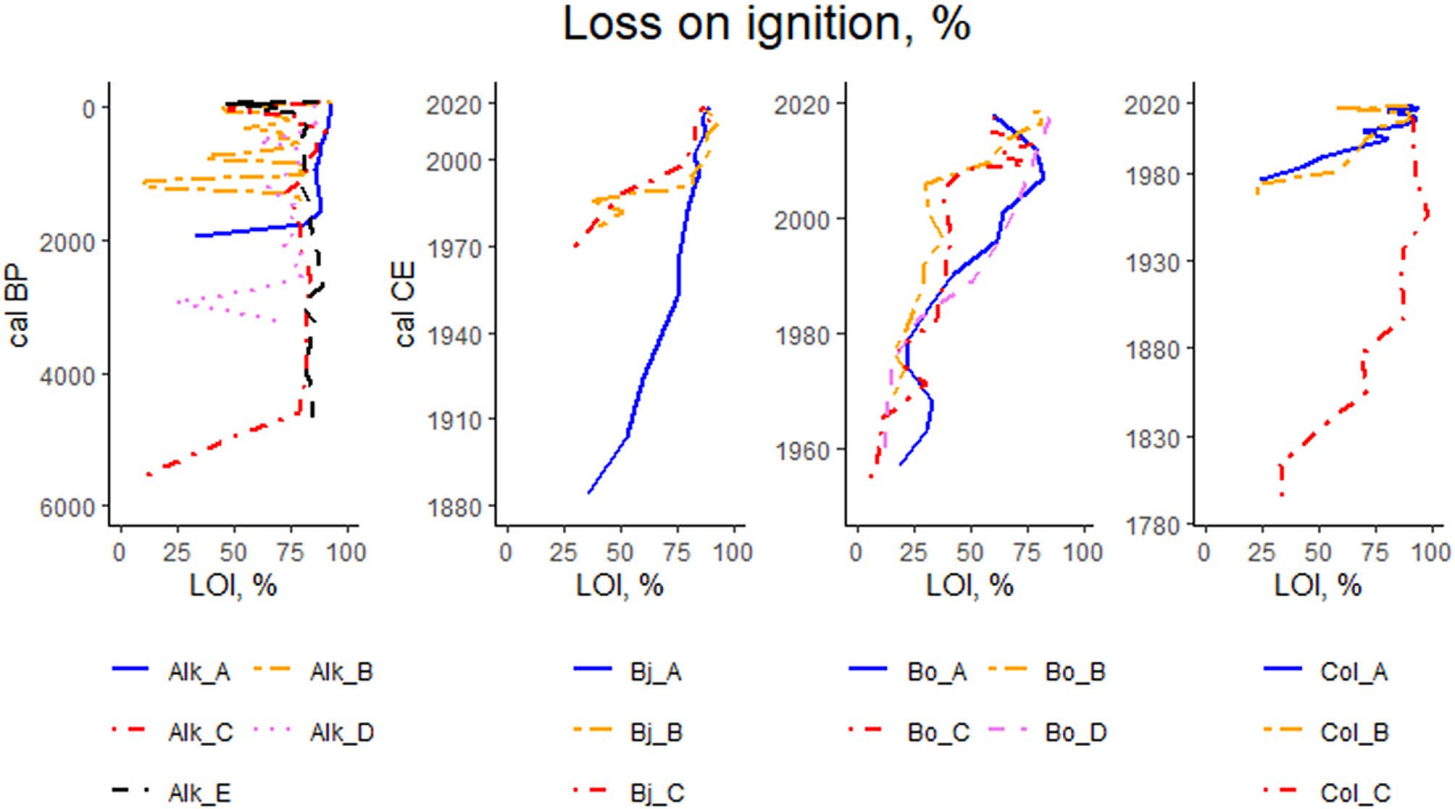
Loss on ignition (%) at different soil layers. Each panel represent different study site. For the study site Alkhornet (Alk), age is presented as calibrated Before Present (cal BP) while for Bjørndalen (Bj), Bolterdalen (Bo) and Colesdalen (Col) ages are presented as calibrated Common Era (cal CE).

Highest LOI values are typically found in the topmost part of soil profiles (Fig. 7). In our study, highest LOI values for each study site are 93.3% (Alk_B), 92.5% (Bj_B), 85.1 (Bo_D) and 98.4% (Col_C). For all of the study points included in this research, the average LOI value with standard deviation (± SD) is 71.4 ± 28.6%. Large fluctuations in LOI at various depths is found in all of study points of Alkhornet study site apart from study point Alk_A (Fig. 7). In study points Bo_A, Bo_C, Col_A and Col_B fluctuations in LOI occur in the surface part of the profile while in study point Bj_B large fluctuation is seen above the mineral soil contact.

**Figure 8:**
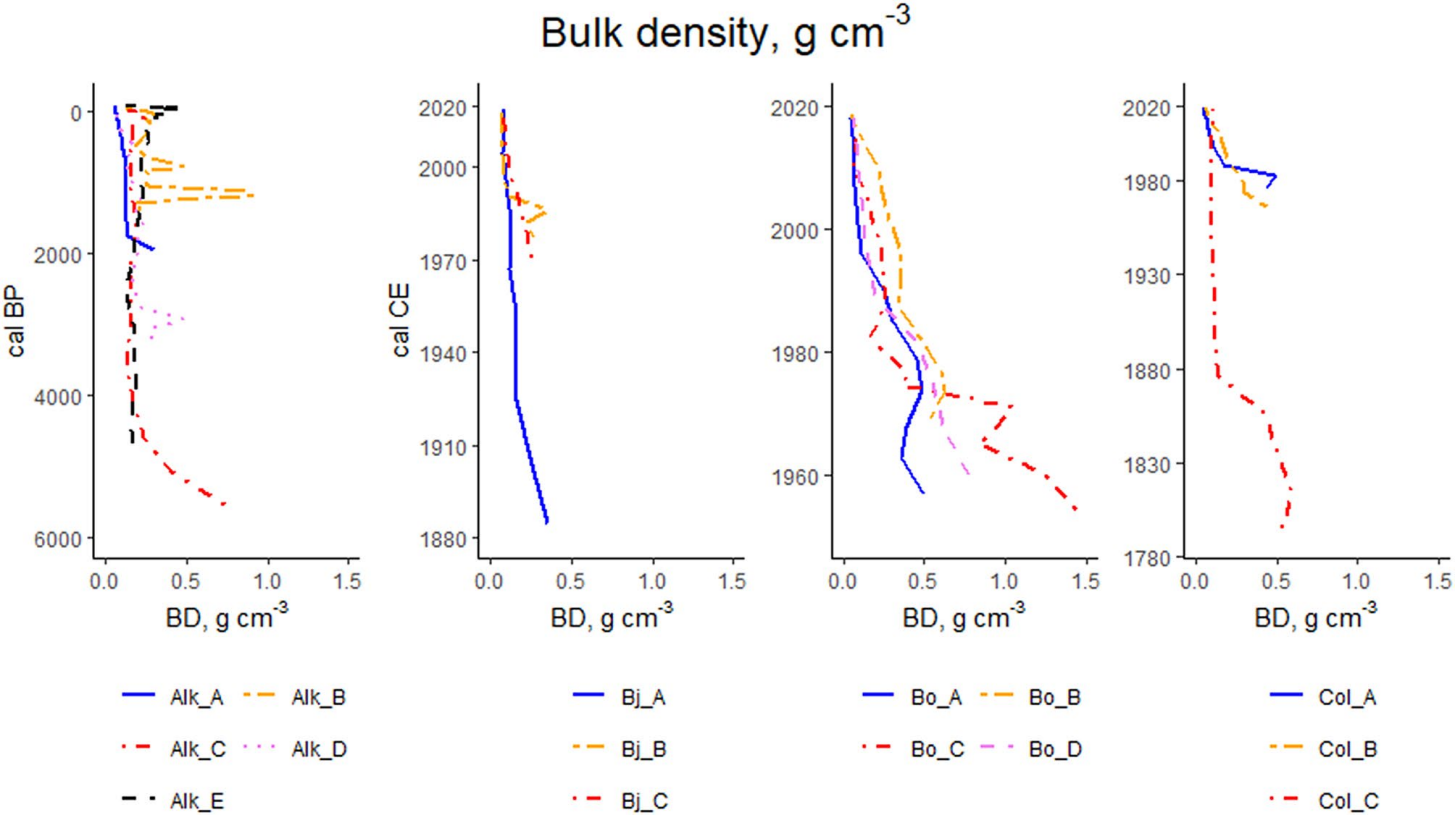
Bulk density, g cm^-3^ at different soil layers. Each panel represent different study site. For the study site Alkhornet (Alk), age is presented as calibrated Before Present (cal BP) while for Bjørndalen (Bj), Bolterdalen (Bo) and Colesdalen (Col) ages are presented as calibrated Common Era (cal, CE).

In general, bulk density values (BD, g/cm^3^) mirror the LOI values for our study sites (Fig. 8). Mean BD for all study points in Alkhornet is 0.22 ± 0.14 g cm^−3^. Mean BD for the younger study points are 0.12 ± 0.07 for Bjørndalen, 0.14 ± 0.09 for Bolterdalen and 0.17 ± 0.16 for Colesdalen. The average BD for all the study points is 0.19 ± 0.13 g cm^−3^. Highest bulk densities in Bjørndalen (0.35), Bolterdalen (0.36) and Colesdalen (0.59) are encountered in the lower part of the soil profiles where mineral content is high (Fig. 8). Similar to LOI values we discovered large fluctuations in BD across the soil profiles in Alkhornet, excluding study point Alk_A. In general, BD values decrease either gradually or sometimes abruptly (Col_A, Col_C) towards the surface.

#### Nitrogen and carbon content

**Figure 9:**
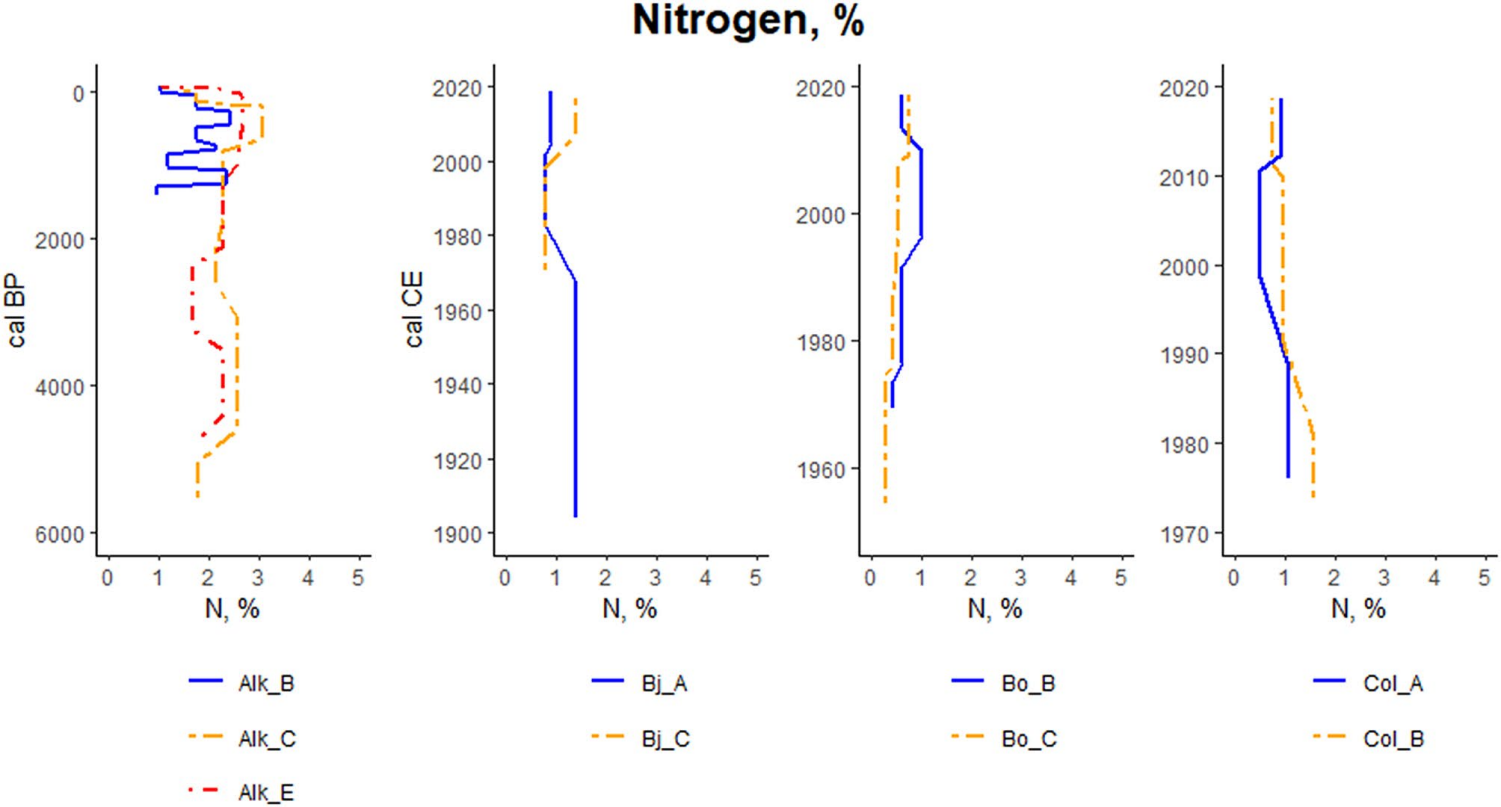
Nitrogen content (%) variation at different soil layers for focus section study points. Each panel represent different study site. For the study site Alkhornet (Alk), age is presented as calibrated Before Present (cal BP) while for Bjørndalen (Bj), Bolterdalen (Bo) and Colesdalen (Col) ages are presented as calibrated Common Era (cal, CE).

**Figure 10:**
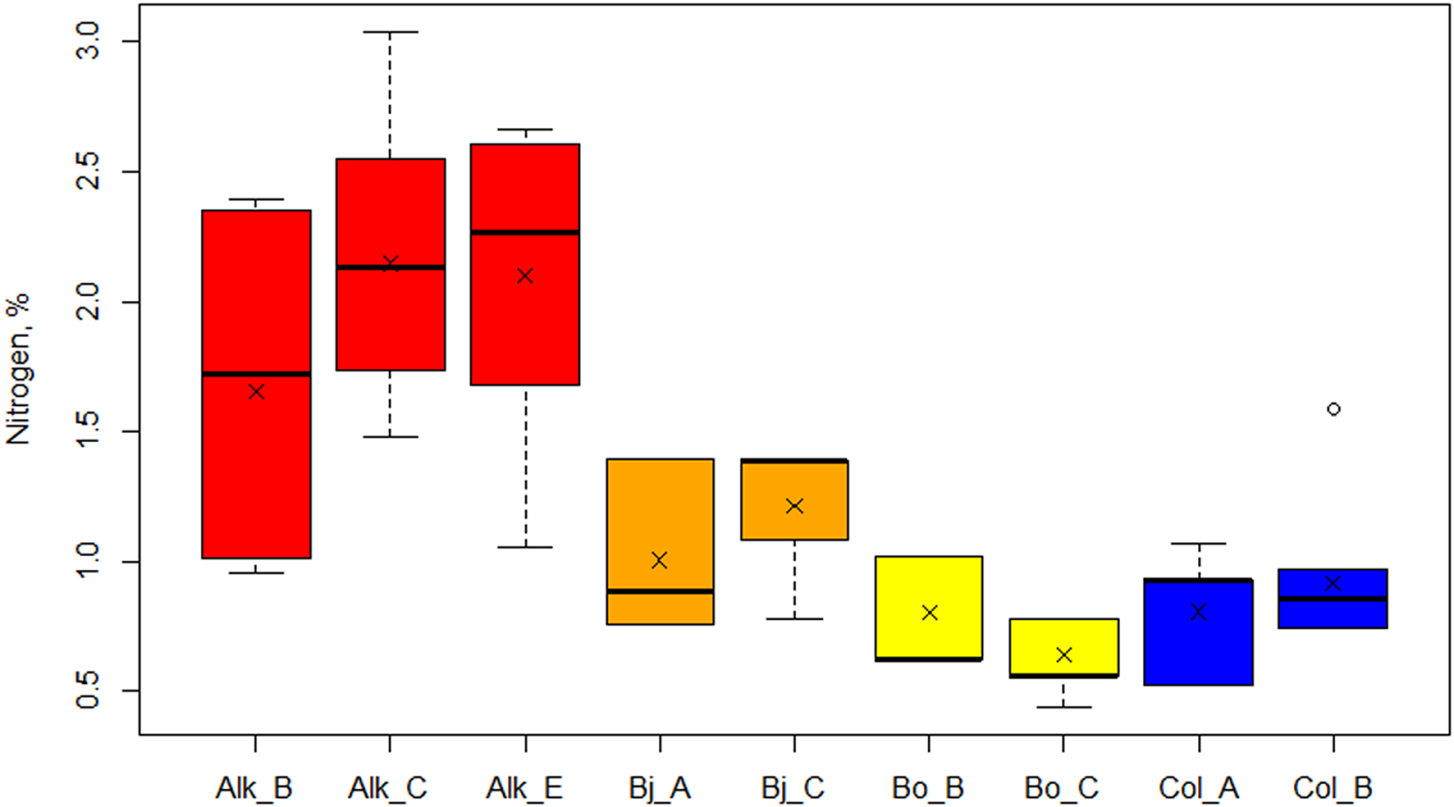
Nitrogen content (%) for the focus section study points. The × marks mean value and the vertical line median value. Where applicable, whiskers represent 1st and 3rd quartile. Outliers are shown as dots outside the boxplot.

For the focus soil profiles, nitrogen contents range from (Bj) 0.8 to 1.4%, (Bo) 0.4% to 1.0% and (Col) 0.5% to 1.6% in the younger sites (Fig. 9). For these locations the mean nitrogen content is (Bj) 1.1 ± 0.3%, (Bo) 0.7 ± 0.2% and (Col) 0.9 ± 0.2% (Fig. 10). In the focus soil profiles of Alkhornet, the nitrogen content is markedly higher than in the other sites, varying from 1.0% to 3.0% while the mean N content is 2.0 ± 0.6%.

**Figure 11:**
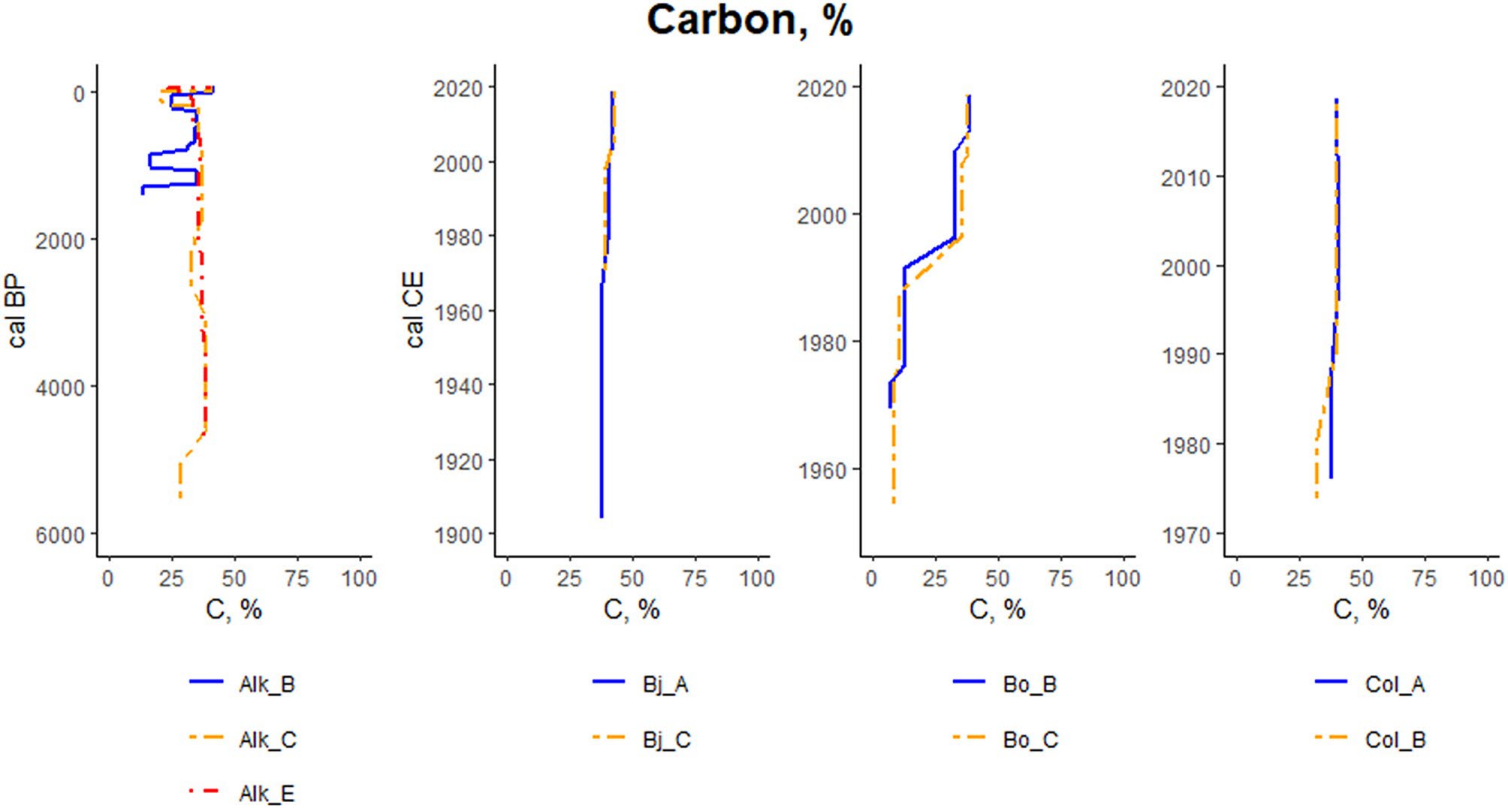
Carbon content (%) variation at different soil layers for focus section study points. Each panel represent different study site. For the study site Alkhornet (Alk), age is presented as calibrated Before Present (cal BP) while for Bjørndalen (Bj), Bolterdalen (Bo) and Colesdalen (Col) ages are presented as calibrated Common Era (cal, CE).

**Figure 12:**
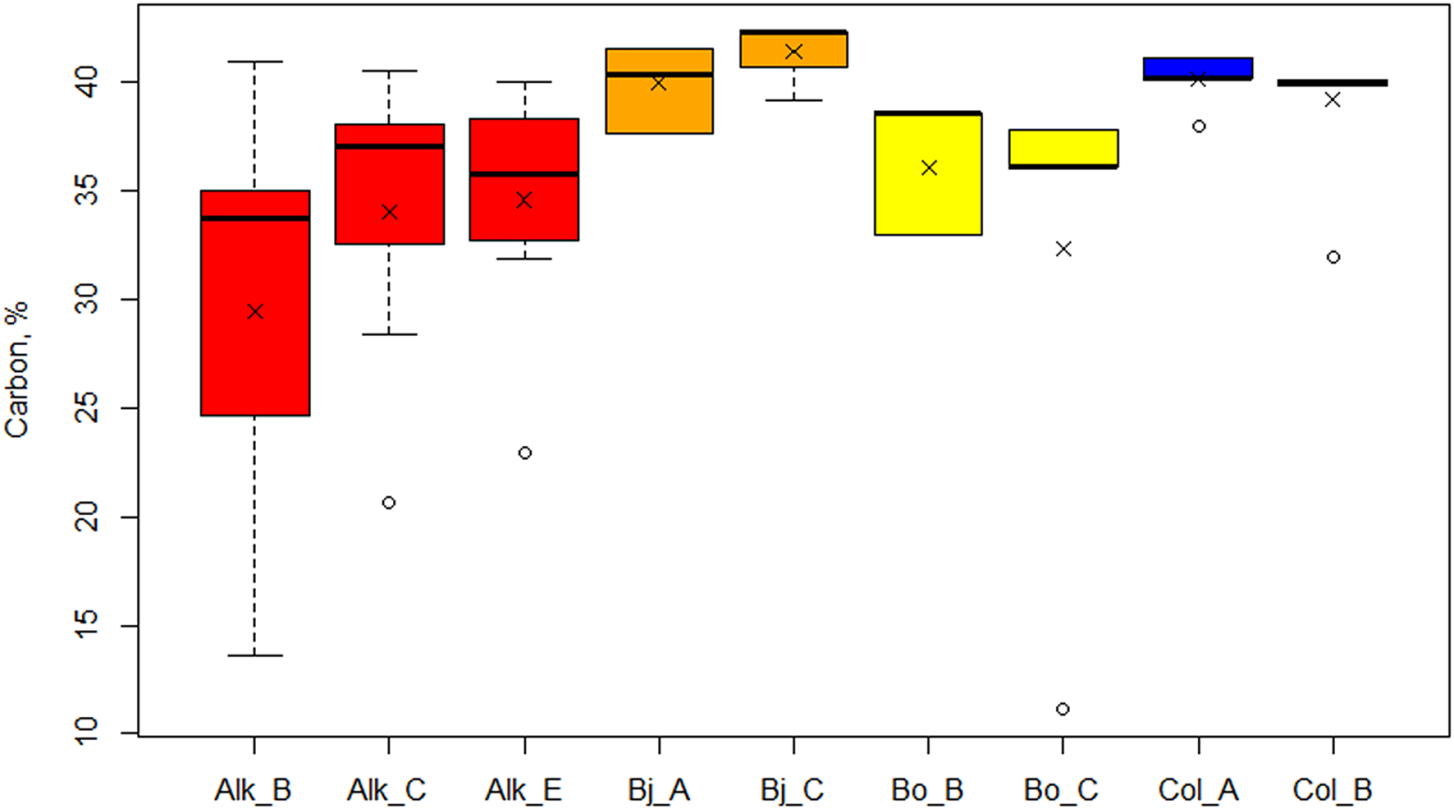
Carbon content (%) for the focus section study points. The × marks mean value and the vertical line median value. Where applicable, whiskers represent 1st and 3rd quartile. Outliers are shown as dots outside the boxplot.

Sample-specific carbon content vary from 37.7% to 42.3% in Bjørndalen, 11.1% to 38.6% in Bolterdalen and 31.9% to 41.1% in Colesdalen. In Alkhornet, the carbon content values range between 13.6% and 40.9% (Fig. 11). The mean carbon content for study sites are (Bj) 40.5 ± 1.7, (Bo) 34.0 ± 8.1, (Col) 39.7 ± 2.0 and 32.7 ± 7.6% (Alk) (Fig. 12).

**Figure 13:**
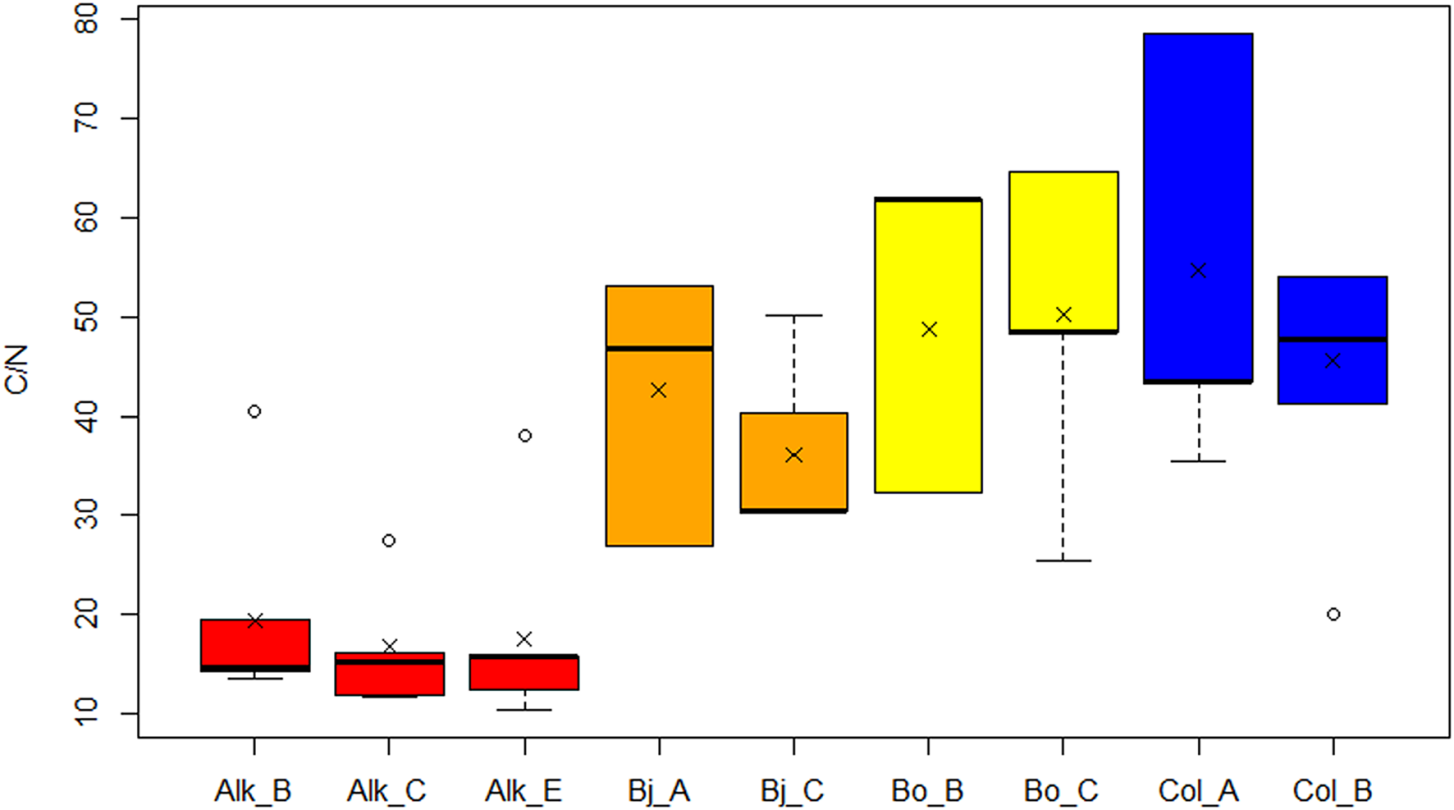
C/N ratio for the focus section study points. The × marks mean value and the vertical line median value. Where applicable, whiskers represent 1st and 3rd quartile. Outliers are shown as dots outside the boxplot.

The C/N values of our younger study sites are noticeable higher than in the Alkhornet study site (Fig. 13). The mean C/N values range from 40.4 ± 10.9 in Bjørndalen, 49.5 ± 14.5 in Bolterdalen and 50.4 ± 16.1 in Colesdalen while in Alkhornet the C/N is 18.0 ± 8.5.

### Organic soil accumulation

The length of the soil profiles varies between 7 and 13 cm in Björndalen, 9–16 cm in Bolterdalen and 11–12 cm in Colesdalen (Table 2). In Alkhornet, where organic soil layer can be considered as mature peat—the thickness being > 30 cm—the length of the soil profiles sampled down to the bottom of the active layer are between 24 and 32 cm. The length of the soil profile Alk_A overlying the mineral soil is 11 cm.

### Carbon storage

**Figure 14:**
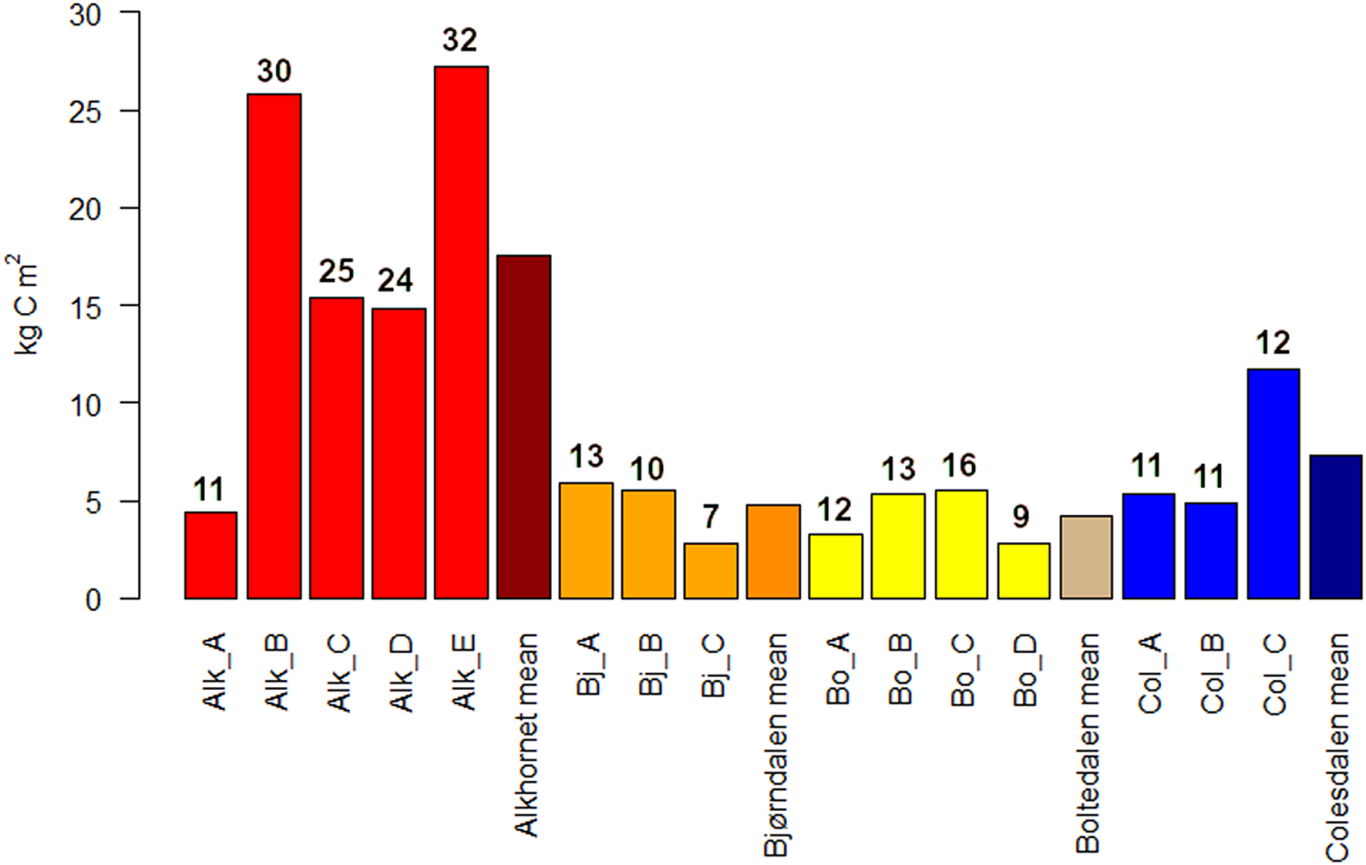
Carbon storage of the study points (kg C m^-2^) measured from the basal layer (Alk_A and study sites Bjørndalen, Bolterdalen and Colesdalen) or from the bottom of the active layer (Alk_B, Alk_C, Alk_D and Alk_E). In addition, mean carbon storage for each study site is included. Values above each bar represent thickness of the organic layer of the study points in cm.

Total carbon storage (g C cm^−2^) measured from the basal layer (study point Alk_A and study sites Bjørndalen, Bolterdalen and Colesdalen) or from the bottom of the active layer (Alk_B, Alk_C, Alk_D and Alk_E) vary from 4.4 (Alk_A) to 27.2 (Alk_E) kg C m^-2^ in Alkhornet (Fig. 14). In Bjørndalen carbon storage is between 2.8 (Bj_C) to 5.9 (Bj_A), in Bolterdalen 2.8 (Bo_D) to 5.6 (Bo_C) and in Colesdalen 4.8 (Col_A) to 11.7 (Col_C) kg C m^-2^.

### Testate amoeba community changes

**Figure 15:**
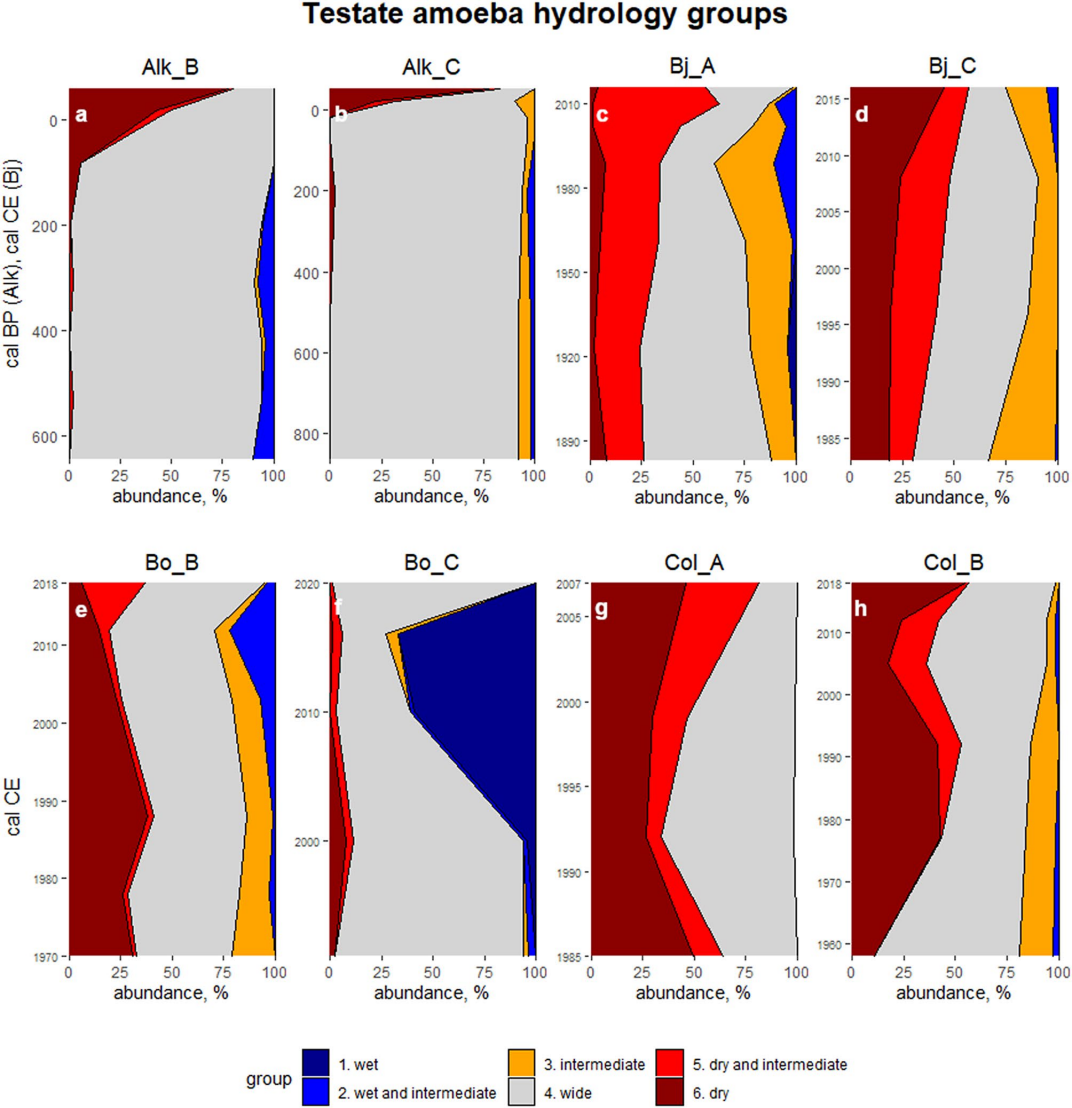
(**a**)–(**h**). Testate amoeba groups (%) by their hydrological preferences for study points Alk_B (**a**), Alk_C (**b**), Bj_A (**c**), Bj_C (**d**), Bo_B (**e**), Bo_C (**f**), Col_A (**g**) and Col_B (**h**). For study site Alkhornet, age is presented as calibrated Before Present (cal. BP) while for the other sites ages are presented as calibrated Common Era (cal CE).

The analysed soil profiles indicate that testate amoeba taxa adapted to dry conditions have been most common over the last decades, but also taxa with wide tolerance are abundantly present (Fig. 15a-h). In Bjørndalen, Colesdalen and Alkhornet, taxa with dry and dry-to-intermediate preferences become more common towards the present time, at the depths of 3–5 cm. Moreover, the study sites where testate amoeba assemblages suggest that wet conditions prevailed previously indicate a drying trend towards current times (Fig. 15e, f). The highest proportional community change towards dry is detected in uppermost 3 cm for Alkhornet profiles (Fig. 15a, b). The start of this change is dated to mid-twentieth century when the proportion of dry taxa gradually rises from < 10% to ca. 80% by the 2010’s.

## Discussion

We studied the initiation and development history of organic soils in areas surrounding the Isfjorden in High Arctic Svalbard. The sites studied represented similar geographical setting, climate regime and vegetation communities, yet the sites evidenced divergent succession histories, where the soil properties, age of the organic soil initiation and later development clearly varied. Our special interest was to investigate if the post-LIA and the current warming, starting from the 1980’s^17,18^, respectively, have accelerated organic soil establishment and accumulation, and consequently enlarged the regional carbon stock. Higher temperatures and increase in air moisture can increase vegetation production in the northern latitudes, although this is also controlled by local conditions^56–58^. Post-LIA glacial retreat driven by warming climate has also exposed new soils, which became available for plant colonization and vegetation that form organic soils^59,60^. In our study, we found old, well-developed peatland site and young sites with only recently initiated organic soil layers. The results from our young study sites show that in addition to primary succession and organic soil development on freshly revealed soils, similar accumulation of organic matter has been happening on sites without evidence of previous glacial cover. This suggests an improvement in growth conditions that has led to an excess biomass production, leading to accumulation of organic soil layers and later lateral expansion of these sites. The persistence of the older peat layers existing in Svalbard and the new, post-LIA organic soil layers shows that the carbon stocks in Svalbard are likely increasing.

### Organic soil initiation and Holocene environmental conditions

Based on the active layer basal age, the initiation of the organic soil accumulation in our oldest study site, Alkhornet, occurred ca. 6000 BP at latest. However, the exact timing of the initiation remained unresolved as we could not penetrate to frozen peat layers, thus the timing of the initiation is likely older. Palaeoecological studies have proven a link between climate and organic soil development patterns. In North American and Eurasian continents, warm and humid early Holocene conditions accelerated peatland initiation as the biomass production was promoted by favourable growth conditions, high effective moisture level in particular^6,8^. During the mid-Holocene (8.2–4.2 ka BP), the climate in Svalbard was warmer and more humid than present-time conditions, with 1–2 °C higher annual average temperatures^9,13^ and organic soil accumulation was already on-going in Alkhornet. High number of peat basal ages dated to the mid-Holocene suggest comparable response of peatland dynamics in Svalbard as for the early Holocene elsewhere in Northern Hemisphere^21,22,61–66^.

The late Holocene climate cooling ca. 4 ka BP onwards, a period known as Neoglacial cooling, ended the productive Holocene era. At the same time, glacial movements increased erosion and intensified gelifluction and solifluction as well as other glacio-fluvial and aeolian processes leading to relocation of mineral soils, which again buried existing peatlands and vegetated habitats^63,64,66–68^. In addition, pollen data from Svalbard suggest unfavourable growth conditions during the late Holocene and this led to reduction of both vegetation cover and biomass density^69^. Subsequent low biomass input to organic soils potentially resulted in either a slowdown or a complete cessation of peat or organic soils growth and development. However, also during the unfavourable Neoglacial conditions, some initiation of organic soils has been reported in Svalbard^22,23,70^. The thick peat layers have been found previously at several sites that have not been affected by soil disturbances, suggesting a continued organic matter accumulation also through the colder climatic conditions^22,61,63,65^. Peat development and lateral expansion continued also in Alkhornet proven by a basal age of 1950 BP dated for the mineral soil-peat contact at the margin of the Alkhornet study site (Alk_A). Even small-scale micro-climatologically suitable conditions are known to increase biomass production, thus enabling local initiation and accumulation of organic soils^71^.

Our thin and young soil profiles reveal that new organic soil initiation has occurred across Isfjorden area in the Svalbard dated to recent decades following the end of the LIA. Growth conditions in Svalbard are gradually approaching those experienced during the mid-Holocene and are accompanied by release of new areas suitable for vegetation colonization due to retreating glaciers. Accordingly, the increase in total vegetation productivity and consequent organic matter accumulation in Svalbard is apparent. Increased ‘greening’ of Svalbard during last 30 years^72^ supports this development pathway. This suggests that growth conditions have improved consequently and enabled accumulation of organic layers in the areas previously not suitable for organic soil accumulation.

The soil profiles collected from the three other study sites (Bjørndalen, Bolterdalen and Colesdalen) suggest recent initiation of organic soil accumulation. However, the lack of older organic soil layers does not exclude the possibility that vegetated surfaces and organic soil layers existed before in these sites. In the 1980’s Låg^61^ reported some 40–80 cm thick, partly buried peat deposits in Bjørndalen but unfortunately these profiles were not dated. Moreover, Surova et al.^64^ discovered a buried peat deposit up to 2.5 m thick near our study site in Colesdalen and the acquired basal age of 4290 ± 45 BP suggest a late Holocene initiation. These deposits were covered by a mineral layer 40 cm thick that was transported to the site by glacial meltwaters, halting the organic material deposition. These data suggest the land areas at the vicinity of our study sites in Bjørndalen and Colesdalen may have had organic soil formation already thousands of years before the initiation of the soil profiles presented in this study. We hypothesize that the previously deposited organic soils have been removed by a local disturbance, or that they are currently still covered by thick mineral soil layers impermeable to the coring equipment used in this study. To confirm these hypotheses, a study including deeper soil layers would be required. In any case, fluctuations in LOI and BD are relatively common phenomena in organic soil layers studied in Svalbard^21,63,73^. Our results and those of other studies suggest that the mineral matter transportation is an ongoing process and transportation of the mineral matter even leading to complete burial of previous soil layers has to be taken into account^62–64,66,67^. However, based on the LOI values of our soil profiles we infer that our study sites have avoided the total burial of organic sediments during their current developmental history.

Contrary to Bjørndalen and Colesdalen, which did not indicate any direct evidence of earlier disturbances, Bolterdalen, with the youngest basal ages of 1955 CE, was likely covered by the Foxfonna glacier during the LIA as shown by a study by Martin-Morena et al.^74^ The Foxfonna glacier retreat exposed the study site following the post-LIA warming^10,75^. The exposed lands enabled vegetation establishment from the early twentieth century on. We suggest that the near simultaneous basal ages of 1955 CE (Bo_A), 1960 CE (Bo_B), 1955 CE (Bo_C) and 1960 CE (Bo_D) reflect contemporaneous retreat of the glacier front, and simultaneous initiation of the organic soil deposits shortly after. A ca. 50-year lag between organic soil initiation and the timing of the retreat of the Foxfonna glacier can be explained by the typical delay in primary succession on the glacial foregrounds. It can take several decades or more after the soil has stabilized and the growth conditions are suitable for vascular plants and bryophytes to colonize and organic soil may start to accumulate^28,60,76^.

### Changes in hydrological conditions

The testate amoeba data indicate a strong, general drying trend for all our study sites. To our knowledge, no such data are available from the Spitsbergen, the main island of the Svalbard. However, our results are supported by data collected from the smaller Edgeøya island in south-eastern Svalbard reconstructing moisture conditions and suggesting drying towards the modern times^77^. These data are controversial to the measured increase in precipitation rates in Svalbard over the last 120 years. Interestingly, one possible explanation for surface drying might be permafrost thaw. Permanently frozen ground can sustain high water table levels by preventing vertical water flow. Instead, thawing permafrost increases drainage, which possibly leads to local surface drying^78^. First signals of permafrost warming have been measured in the Svalbard^79^, although it should be noted that in mountainous regions permafrost conditions are difficult to assess due to the large heterogeneity of the landscape^80^. Another possible factor contributing to surface drying is glacial retreat induced by recent warming. The glacial retreat initially increases the amount of meltwater discharge but later, changes in the position of the glacial front may lead to diverged meltwater channels resulting in local losses of moisture input and desiccation^21^. Based on the CryoClim data set of Svalbard glaciers^81^, shrinkage of the glaciers nearest to our study sites has been observed. However, glaciers still remain within the watershed area of Bjørndalen and Bolterdalen, and these sites have also suffered drying. Thus, although the retreat of the glaciers may induce drying of organic soil surfaces, explanation is not exhaustive. In addition, increase in temperatures may lead to enhanced evapotranspiration leading to surface drying if this is not compensated by increased precipitation inputs^82^. Regardless of the ultimate reason behind the reconstructed drying trend, the long-term consequences for the biomass production and organic soil accumulation may be severe as drying may decrease carbon accumulation and cause losses of old carbon stored in organic soils^5,83^.

### Impact of local fauna on soil development history

Various studies have reported the fertilizing effects of bird colonies in organic soil layer in the High Arctic^25–27^. Our finding from the Alkhornet study site supports these previous studies. The Alkhornet study site located below a large bird colony shows nearly twice as high nitrogen content compared to the three other sites without bird colonies present. The presence of bird colonies with high inputs of nitrogen fertilization have been recognized as an important driver behind initial development of organic soils^24,28^ and they are commonly found at the vicinity of thick peat soils similar to Alkhornet. Moreover, N content of our thin and young profiles was also comparably high^84^. This may reflect presence of the Svalbard reindeer, which is known to affect the nutrient status and vegetation composition by grazing and fertilization^29–31^. During the field campaign, high amounts of reindeer droppings was frequently detected on the ground.

### Arctic organic soil carbon

Comparison between carbon storage values between different studies remains challenging due to large heterogeneity in landscapes as well as different methods used for sampling and generalization in carbon stock upscaling. Most studies report carbon storage for the top 30 cm or the top 100 cm of the soil profile. As such, our results from the younger sites with depth of 7–16 cm are not directly comparable to these results, while the Alkhornet mean peat depth of 24.4 cm enables approximate comparison. Hugelius et al.^85^ reported a carbon stock of 9.8 ± 7.4 kg C m^-2^ for the top 30 cm soil profiles for the High Arctic globally while values of 7.5 ± 3.3 kg C m^-2^ were found in High Arctic Greenland^86^ and 10.3 ± 4 kg C m^−2^ in the Siberian arctic tundra^87^. While taken into account the relatively young age of the current sites, the measured carbon stock values are of similar size or larger than those found in the aforementioned studies.

Based on thin organic layer values previously reported from Svalbard, our study sites appear to be hot spots for organic matter accumulation. Considering the thickness of the collected soil profiles, our results from Bjørndalen (4.8 kg C m^−2^), Bolterdalen (4.2 kg C m^−2^), Colesdalen (7.3 kg C m^−2^) and Alkhornet (17.5 kg C m^2^) show high carbon stocks with only the lushest sites in Svalbard having similar values. For example, Wojcik et al.^88^ reported carbon stock values for 30-cm soil profiles for different land-cover classes including fen tundra (15.6 kg C m^−2^), and moss tundra with different vegetation cover (4–1.6 kg C m^−2^). Yoshitake et al.^89^ found carbon stocks of 4.3 kg C m^−2^ (1.1–7.9 kg C m^−2^) down to a depth of 100 cm for pro-glacial foregrounds representing a late succession stage. The differences found between our results and the values reported by Wojcik et al.^88^ and Yoshitake et al.^89^ can be explained by the lack of thick organic soil layers in their studies. The former reported that only the fen tundra had relatively thick organic layer (20 cm) while less thick organic layer of 1.7–2.1 cm were typical for different moss tundra types. The latter found organic soil layer of 2.6–3.1 cm for the sites representing later succession stages. Apart from the fen tundra, these are markedly smaller than the thickness of organic soil profiles (7–32 cm) collected in our study sites. Nakatsubo et al.^23^ measured carbon storage of 4.5 to 9.2 kg C m^-2^ from Stuphallet, ca. 100 km north of our study sites. This value is noticeable smaller than carbon storage calculated for Alkhornet, which has similar site characteristics: a nearby bird colony and a thick organic layer. In addition, our sampling methods are comparable. The difference in carbon stocks found between Alkhornet and Stuphallet is most likely explained by higher degree of decomposition and mineral matter mixed in our soil profiles, which can be seen in the BD values that are over twice as high as those measured by Nakatsubo et al.^23^

Our results reveal locally high carbon stocks for the High Arctic. In addition to sites already supporting thick organic soil layers, such as Alkhornet in this study, sites similar to those of Bjørndalen, Bolterdalen and Colesdalen have potential to become important carbon sinks if the predictions of improving growth conditions in future actualise. However, the changing climate may also cause loss of carbon from the organic soils if, for instance, tundra fire frequency increases^90^, permafrost thaw accelerates^5^ and/or if large-scale changes in the vegetation community structure occurs^91^.

## Conclusions

Our results show that large heterogeneity in organic soil accumulation occurs even within a relatively small area, even though some general trends were detected. By only concentrating on a single site, these variations could have been missed, highlighting the importance of high number of replicate measurements and various scales and methodologies to catch the large variety found in these natural environments.

We found organic soil layers from Svalbard that have initiated only recently, over the last decades and after the Little Ice Age. In Bolterdalen study site, this recent initiation is connected directly to a retreat of the nearby glacier. However, in Bjørndalen and Colesdalen no such a link could be established. Recent initiation of organic soil layers likely reflects improved climatic conditions and/or stabilization of the underlying mineral substrate. Regardless of the underlying cause, the recent initiation of organic soils accumulation suggests establishment of potentially important new carbon sink in the High Arctic. Compared to previous studies presenting carbon stocks for the Arctic areas, our soil profiles suggest effective carbon sink capacity and should be taken into account in High Arctic carbon budget estimations. Decrease in effective moisture level in the future may endanger accumulation of newly initiated organic soils. In contrast to weather data suggesting increase in precipitation, our proxy data suggest a drying trend across all of the sites studied probably driven by glacial processes. If continued, such development would also affect existing and future carbon stocks. Palaeoecological data of high arctic organic soil processes in Svalbard and elsewhere in High Arctic are still scarce. Thus, further research on the newly initiated organic soil layers is needed to reveal their total importance on the High Arctic carbon stocks.

## Supplementary Information

Supplementary Information.

## Data Availability

Data used in the study is available from the corresponding author on a reasonable request.
