# Author Correction: Newly initiated carbon stock, organic soil accumulation patterns and main driving factors in the High Arctic Svalbard, Norway

**DOI:** 10.1038/s41598-023-30353-0

**Published:** 2023-03-02

**Authors:** T. Juselius, V. Ravolainen, H. Zhang, S. Piilo, M. Müller, A. Gallego-Sala, M. Väliranta

**Affiliations:** 1 Environmental Change Research Unit (ECRU), Ecosystems, Environment Research Programme, Faculty of Biological and Environmental Sciences, and Helsinki Institute of Sustainability Science (HELSUS), University of Helsinki, Viikinkaari 1, P.O. Box 65, 00014 Helsinki, Finland; 2 Fram Centre, Norwegian Polar Institute (NPI), 9296 Tromsø, Norway; 3 Key Laboratory of Cenozoic Geology and Environment, Institute of Geology and Geophysics, Chinese Academy of Sciences, Beijing, China; 4 Department of Geosciences and Geography, Faculty of Science, University of Helsinki, Yliopistonkatu 3, P.O. Box 4, 00014 Helsinki, Finland; 5 Geography, College of Life and Environmental Sciences, University of Exeter, Exeter, UK

Correction to: *Scientific Reports* 10.1038/s41598-022-08652-9, published online 18 March 2022

The original version of this Article contained errors in Figure 2 and 4, where incorrect versions of these figures were published. The original Figure 2 and 4 and their accompanying legends appear below.

**Figure 2:**
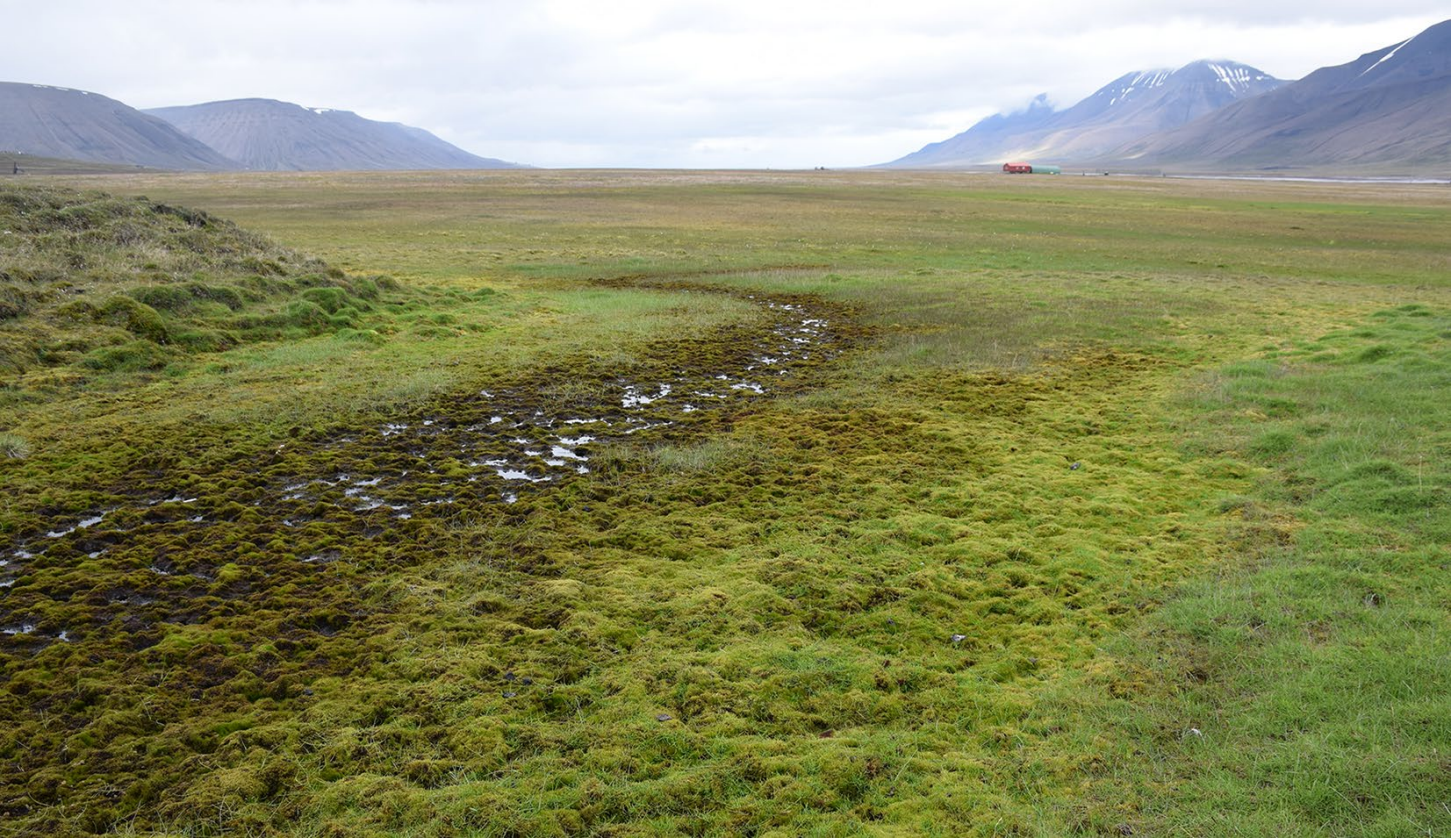
The general view of the Bjørndalen study site. The variations at the local scale can be seen in wet depression with mosses and dry surfaces with grass species.

**Figure 4:**
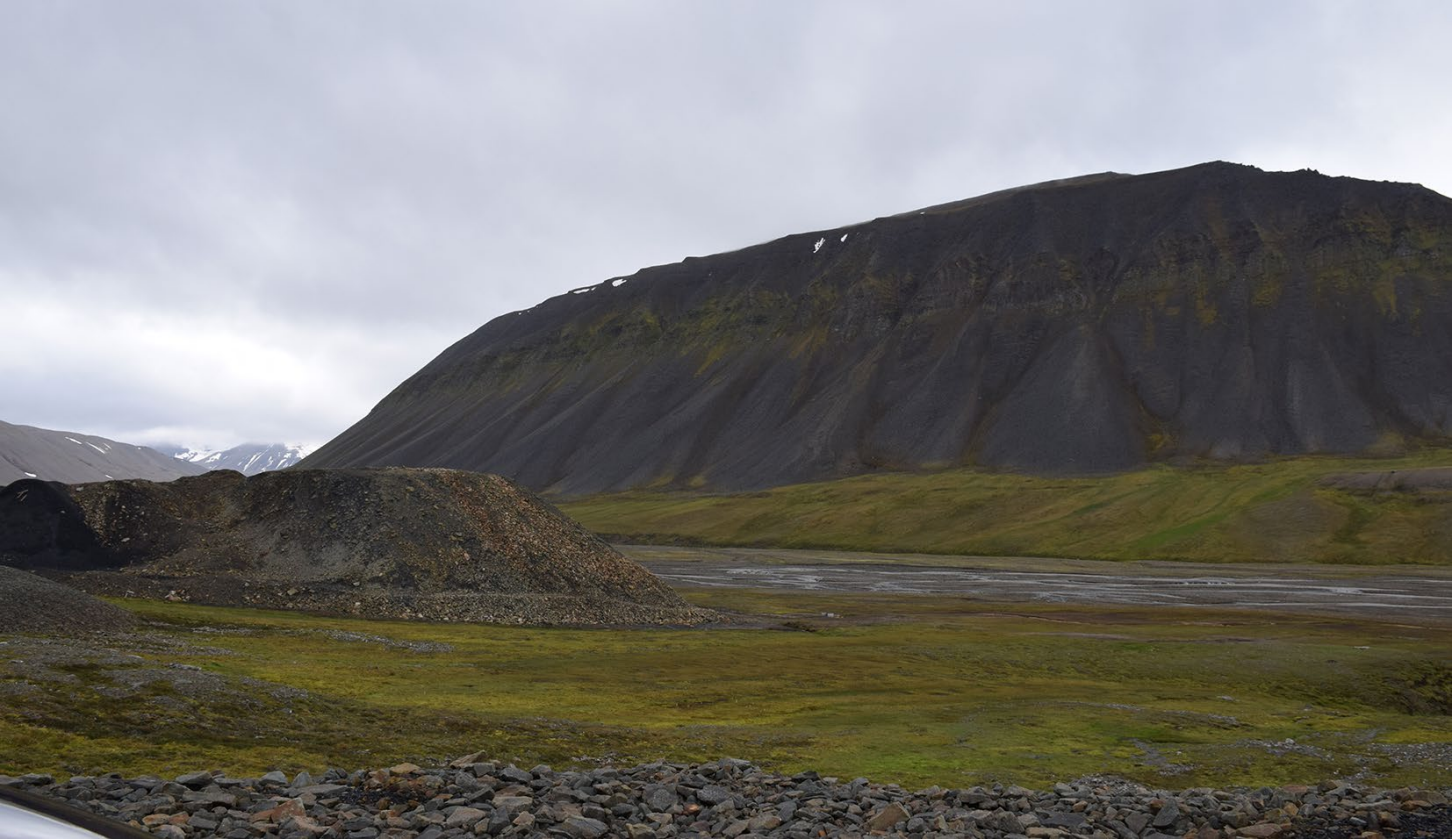
The general view of the Colesdalen study site. Vegetation and microforms in Colesdalen are similar to those of the Bjørndalen and Bolterdalen. In the front and at the back of the image non-vegetated mineral disturbance surfaces can be seen.

The original Article has been corrected.

