# Supplementary material for: Newly initiated carbon stock, organic soil accumulation patterns and main driving factors in the High Arctic Svalbard, Norway
Source: Sci Rep. 2022 Mar 18;12:4679. doi: 10.1038/s41598-022-08652-9 (PMC8933566; doi:10.1038/s41598-022-08652-9)
Supplement: Supplementary file 1 — Supplementary Information. [file 41598_2022_8652_MOESM1_ESM.pdf]

Newly initiated carbon stock, organic soil accumulation patterns and main driving factors in the High Arctic Svalbard, Norway

Juselius, T.[1*], Ravolainen, V.[2], Zhang, H.[1,3], Piilo, S.[1], Müller, M.[4], Gallego-Sala[5], A and Väliranta, M.[1]

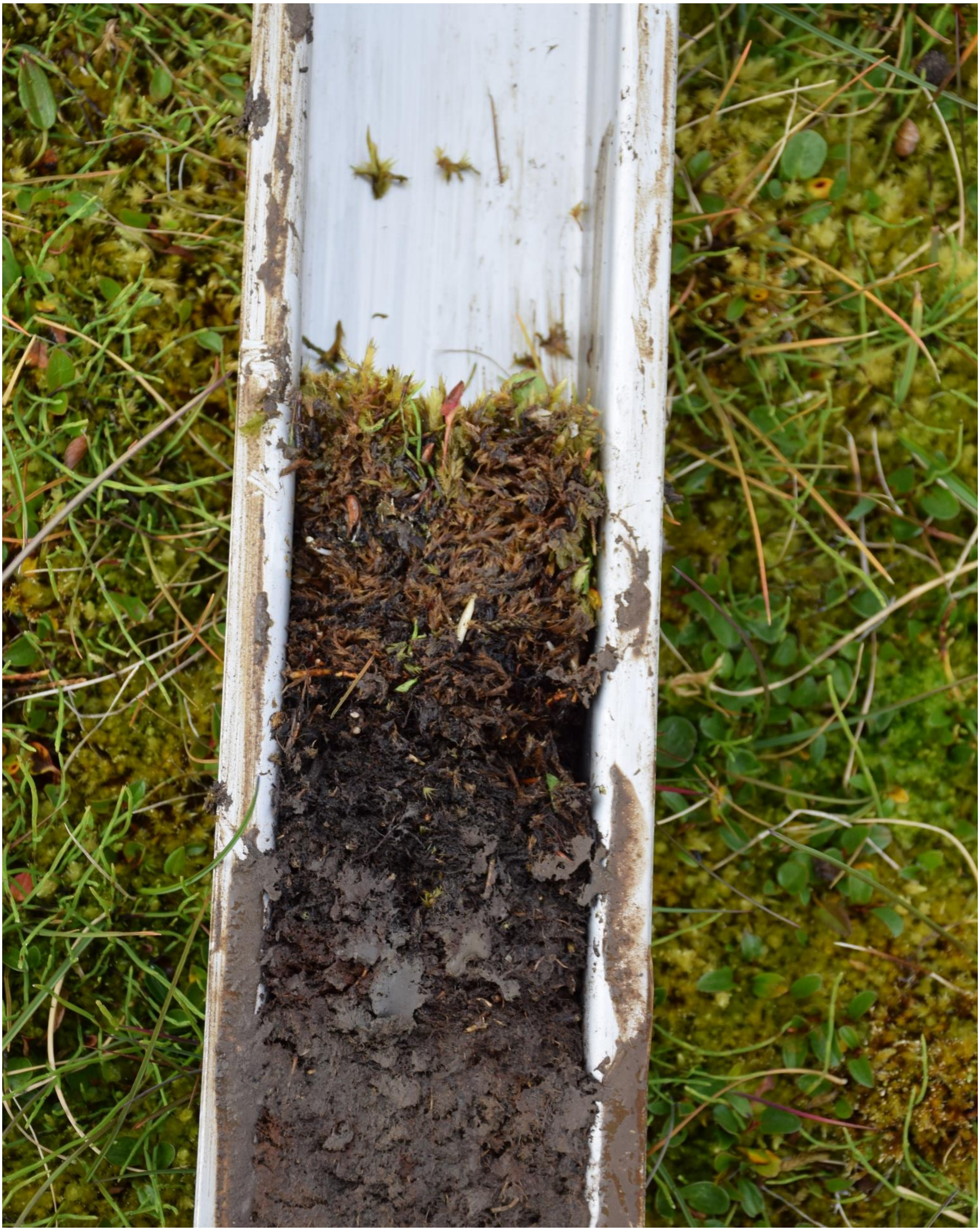

Fig. S1. Example soil profile from study site Bjørndalen.

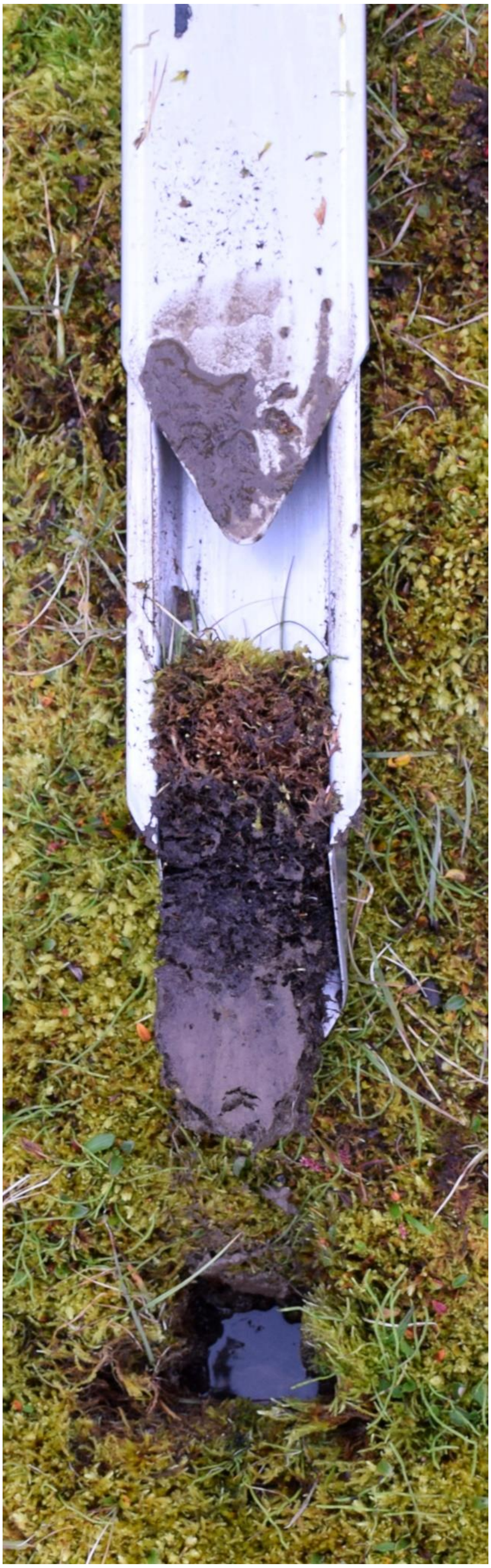

Fig S2. Example soil profile with water table shown from study site Colesdalen.

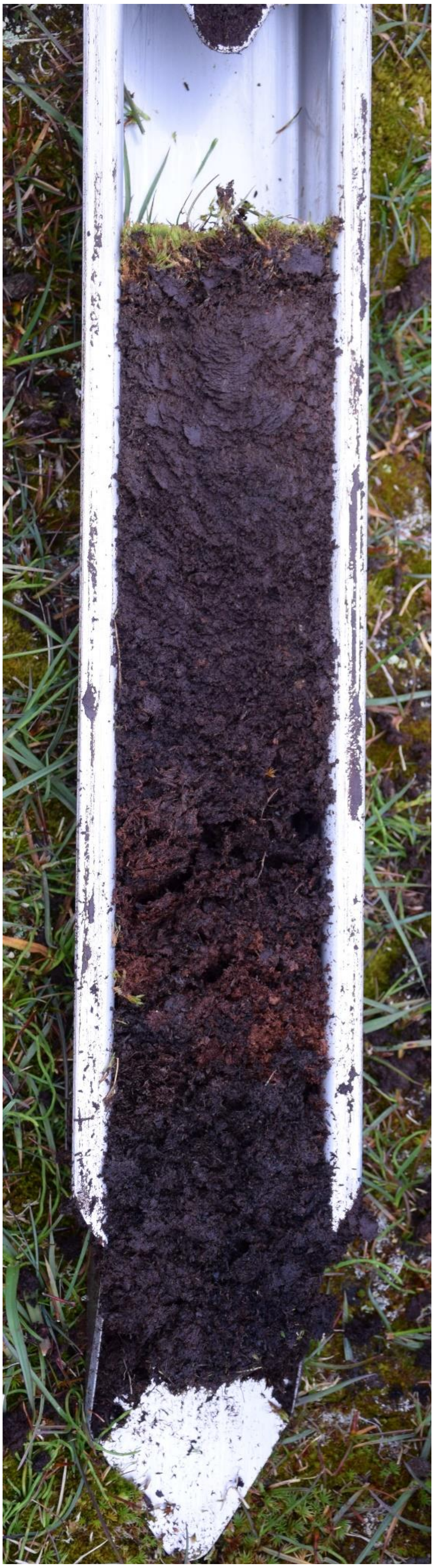

Fig S3. Example soil profile 1 from study site Alkhornet.

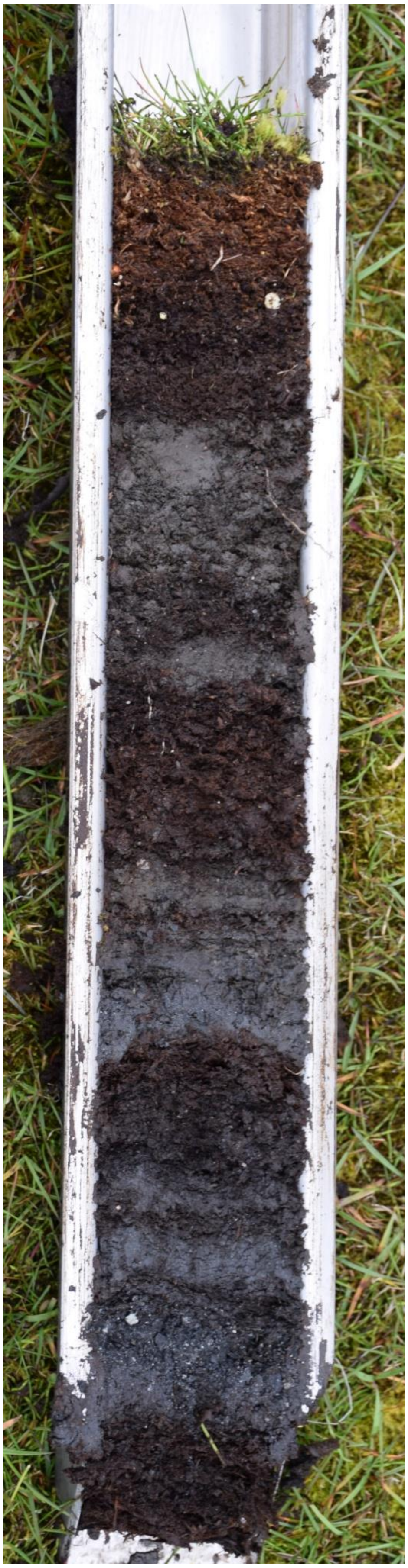

Fig S4. Example soil profile 2 from study site Alkhornet with clear mineral stripes at various depths.

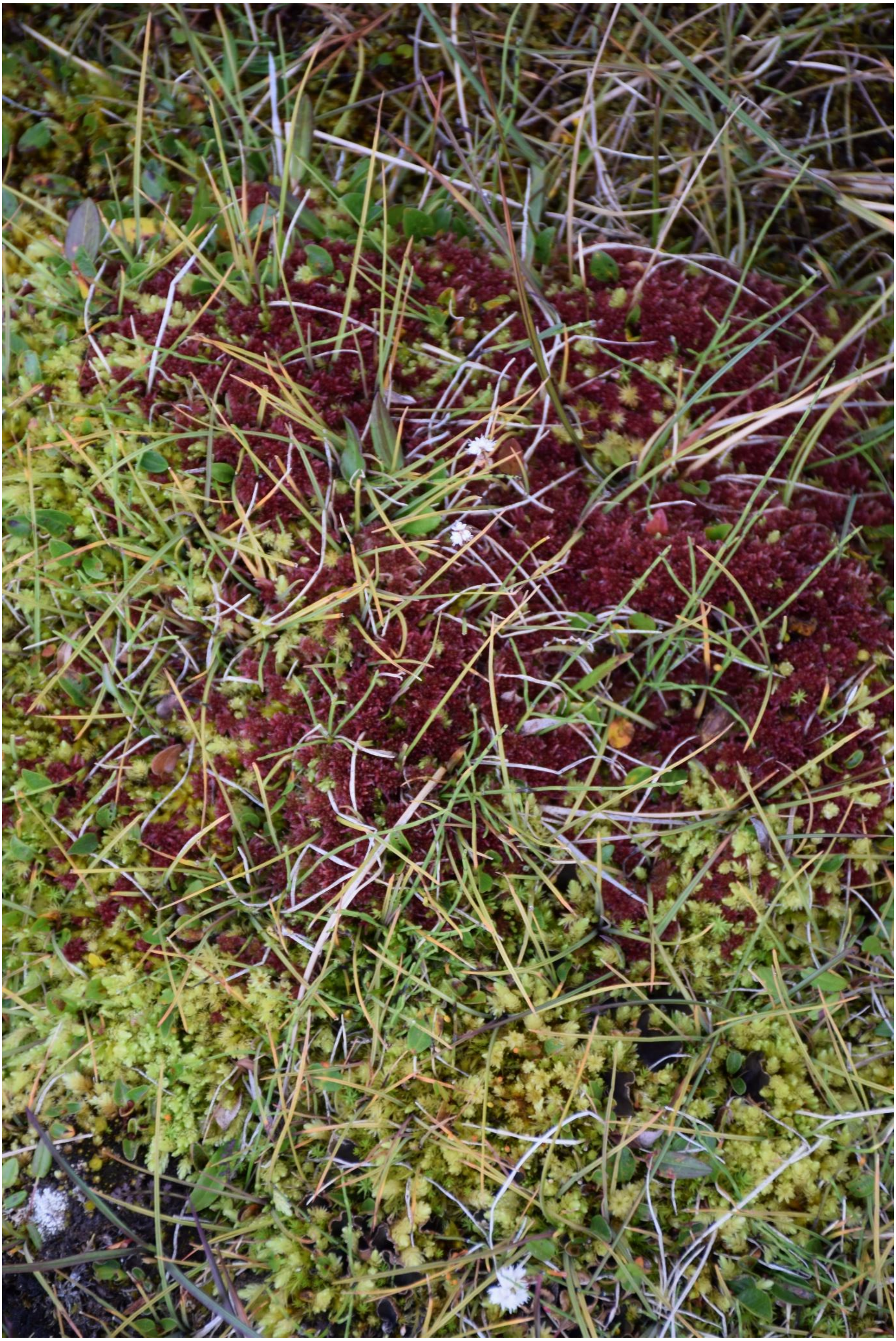

Fig S5. Example figure presenting *Sphagnum* mosses present at the Colesdalen study site.

Supplementary data 1:

MAIN VEGETATION SPECIES OF STUDY SITES

Tomentypnum nitens

Aulacomnium palustre

Equisetum arvense spp. alpestre

Sanionia uncinata

Salix polaris

Bistorta vivipara

Petasites frigidus

Dupontia fisheri

Dryas octopelata

Saxiafraga hirculus

Eriopohorum scheuchzeri

Cassiope tetragona

Poa alpina

Deschampsia alpina
